# Child mental health and income gradient from early childhood to adolescence: Evidence from the UK

**DOI:** 10.1016/j.ssmph.2023.101534

**Published:** 2023-10-14

**Authors:** Murong Yang, Claire Carson, Cathy Creswell, Mara Violato

**Affiliations:** a Health Economics Research Centre, Nuffield Department of Population Health, University of Oxford, OX3 7LF, UK; b National Perinatal Epidemiology Unit, Nuffield Department of Population Health, University of Oxford, OX3 7LF, UK; c Department of Experimental Psychology, University of Oxford, OX2 6GG, UK

**Keywords:** Child mental health, Income gradient, Health inequalities, Internalising problems, Externalising problems

## Abstract

**Background:**

Children from low income families are likely to have poorer mental health than their more affluent peers. However, it is unclear how this association varies at different developmental stages and what the potential underpinning mechanisms are. This study investigates the relationship between family income and mental health problems from early childhood to adolescence in the UK, and examines the potential mediating role of family-related factors over time.

**Methods:**

Data were drawn from the UK Millennium Cohort Study at ages 3, 5, 7, 11, 14 and 17 years. Child mental health was measured by the Strengths and Difficulties Questionnaire Total Difficulties Score, and the Internalising and Externalising subscales. Family income was operationalised as permanent income. Cross-sectional analyses were conducted at each age to examine the association between income and mental health problems, and to examine potential mechanisms based on the Parental Stress and Parental Investment theories.

**Results:**

The samples included 8096 children aged up to 14 years, of which 5667 remained in the study at age 17. Results indicated a statistically significant association between lower family income and poorer mental health in all age groups after adjusting for confounding factors. The strength of the association was reduced after adjustment for Parental Stress and Parental Investment factors, with the larger attenuation driven by Parental Stress factors in most cases. Fully adjusted models suggested an increased independent association between maternal psychological distress and children's mental health as children grew older.

**Conclusions:**

While lower family income is associated with a child's poorer mental health, much of this association is explained by other factors such as maternal psychological distress, and therefore the direct association is relatively small. This suggests that policies targeting income redistribution may reduce child mental health problems, and also benefit the wider family, reducing the prevalence of other associated risk factors.

## Highlights

• A significant association between lower family income and poorer mental health was observed in childhood and adolescence.
• The strength and magnitude of the association was reduced after adjustment for Parental Stress and Investment factors.
• The larger attenuation was driven by Parental Stress factors in most cases.
• An independent association between maternal depression and child mental health increased as children grew older.

## 1 Introduction

There is widespread evidence of the positive association between socio-economic status (SES, including education, occupational status and income) and health. People with higher SES are more likely to have better health than those with lower SES, such as less cardiovascular disease (Kaplan & Keil, 1993), lower mortality rate (Dorling et al., 2007), less psychological distress (Bradshaw & Ellison, 2010), and better self-assessed general health (Humphries & Van Doorslaer, 2000). Studies have found that socioeconomic inequalities in health originate in childhood, and that the impact of family income on child development drives much of the disparity (Case et al., 2002; Currie & Stabile, 2003).

There is a growing interest in the relationship between family income and child mental health, which is a fundamental aspect of child development. Research suggests that children living in low-income families are at a higher risk of experiencing mental health problems. The child mental health-income gradient has been found using data from Denmark (Pryor et al., 2019), Australia (Khanam et al., 2020), Norway (Zachrisson & Dearing, 2015), Germany (Herrmann et al., 2018) and the US (Yeung et al., 2002). Notably a strong link has been found between lower family income and both higher internalising problems (e.g. anxiety or depression) and externalising problems (e.g. hyperactivity or conduct disorder) (Fletcher & Wolfe, 2016; Spence et al., 2002). In the UK, the gradient has been consistently demonstrated in childhood (Holmes & Kiernan, 2013) and adolescence (Collishaw et al., 2019; Lai et al., 2019; Langton et al., 2011) separately. Existing studies examined the association between family income and either overall mental health (Collishaw et al., 2019; Holmes & Kiernan, 2013), or a specific aspect of mental health such as internalising (Langton et al., 2011) and externalising problems (Lai et al., 2019). Most studies have also focused on a specific age group or one aspect of child mental health, and there is limited evidence regarding the association between family income and overall and distinct aspects of child mental health problems, and on whether the associations vary at different stages of child development. A recent study investigated the longitudinal association between family income and overall child mental health problems, internalising, and externalising problems for UK children aged 3–11 years and found that lower absolute family income was associated with worse mental health problems for all three mental health outcomes (Garratt et al., 2017). However, the study by Garratt et al. (2017) did not capture the majority of adolescence, a vulnerable period for mental health problems that needs great attention (Currie, 2020). It also did not consider the potential mediating mechanisms that may explain the association.

It is plausible that a number of factors may underlie the association between family income and child mental health, and that the effect of these may change as the child grows up. Some economic studies use the ‘Parental Investment theory’ (PI) to explain the mediating mechanism underpinning the association (Becker & Tomes, 1986; Mayer, 1997), reflecting parents' ability to invest resources to improve their children's health and development. Families with higher income are able to invest more resources for their children than low-income families, such as better childcare and spending quality time in activities with their children, which in turn may have a positive impact on child development (Mayer, 1997). Another potential pathway underlying the effect of income on child health and development has been proposed by theories outside economics, namely the ‘Parental Stress theory’ (PS), which illustrates how income affects children through parents' non-monetary capacities (Conger & Elder, 1994; McLoyd, 1990; Yeung et al., 2002). Living in poverty leads to a stressful home atmosphere, and parents are more likely to have poor physical and mental health (Yeung et al., 2002). This, in turn, may affect parents' behaviour in relating to their children (increasing negative parenting practices), which ultimately has a negative effect on their children's outcomes (McLoyd, 1990; Yeung et al., 2002). The important role of some PI and PS mechanisms underpinning the child mental health-income gradient has been previously explored by Violato et al. (2011) and Noonan et al. (2018) using data from the UK when children were aged 3–5 years and 11 years, respectively. However, there is limited evidence examining the mechanisms from early childhood to adolescence. The important risk factors underlying the mechanisms may also differ for internalising and externalising problems. For example, postpartum depression was found to partially explain the association between family income and externalising problems in early childhood in a sample of 664 children aged 5 years in the UK, while this did not hold for internalising problems (Rutherford et al., 2019). It is therefore important not only to understand potential mechanisms through which family income affects child mental health in general in childhood and adolescence, but also how they vary across distinct aspects of child mental health, so that targeted clinical/policy strategies to address those can be devised.

This study aimed to investigate the relationship between family income and child mental health in childhood and adolescence, using data from the UK Millennium Cohort Study. The first objective was to investigate whether a significant association between low family income and poorer mental health existed when children were aged 3, 5, 7, 11, 14 and 17 years. The second objective was to explore the potential mechanisms underpinning the observed association. This study contributes to a comprehensive data analysis of the relationship between family income and overall and distinct aspects of child mental health using consistent cohorts in their different developmental stages.

## 2 Methods

### 2.1 Conceptual framework

The study draws on the framework of the health production function (Grossman, 1972) and the extended child health production function (Jacobson, 2000), according to which children are born with an initial health endowment, and their current and future health is the product of parental input and other factors. The empirical model can be written as follows:

(1) $$MH_{it}=\alpha +\beta lnY_{it}+\gamma H_{0}+\delta X_{it}+\varepsilon_{it}$$

where ${MH}_{it}$ is child *i*'s mental health status at time *t*; Y is family income; $H_{0}$ is a set of initial endowments that do not change with time; X is a set of covariates referring to family and child characteristics, and $\varepsilon_{it}$ is the error term.

**Fig. 1:**
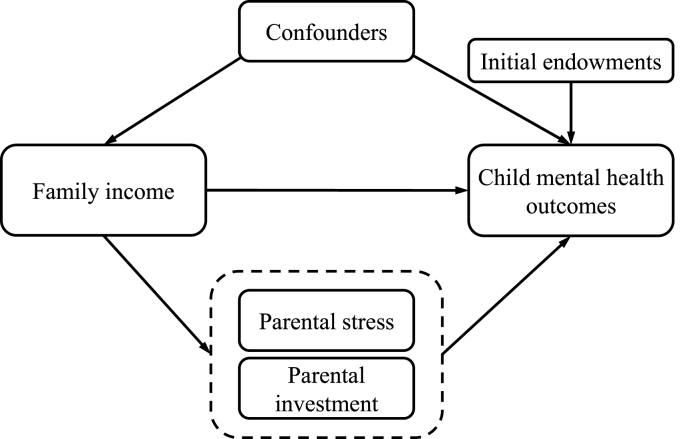
The mechanisms through which income affects child mental health.

Some of the observed associations between family income and child mental health could be partly explained by confounding factors (Fig. 1). Parental stress and parental investment variables may explain the mechanism underpinning the association. By enriching the health production function with the PI and PS theories, Equation (1) can be written as follows:

(2) $$MH_{it}=\alpha +\beta lnY_{it}+\gamma H_{0}+\gamma_{c}C_{it}+\sum_{z=1}^{Z}\gamma_{PS}^{z}PS_{it}^{z}+\sum_{j=1}^{J}\gamma_{PI}^{j}PI_{it}^{j}+\varepsilon_{it}$$

where $X_{it}$ in Equation (1) extends to include a set of confounding factors $C_{it}$, and potential mediating factors with a set of z parental stress variables $\left\{{PS}_{it}^{1},\ldots ,{PS}_{it}^{z}\right\}$ and a set of j parental investment variables $\left\{{PI}_{it}^{1},\ldots ,{PI}_{it}^{j}\right\}$ in Equation (2).

### 2.2 Data and study population

The Millennium Cohort Study (MCS) is a nationally representative longitudinal study of 18,818 children (from 18,552 families) born in the UK in 2000–2002 (Hansen, 2014). It captures a multiplicity of information, including data on children and parents’ health, parental wealth, education, employment as well as other family circumstances and lifestyles (Hansen, 2014). The survey design oversampled children living in the devolved nations, and areas of deprivation with high ethnic minority populations in England (Hansen, 2014). This paper used data from all available surveys, when children were aged 9 months, 3, 5, 7, 11, 14 and 17 years old (University of London, 2017a, 2017b; 2017c; 2017d; 2017e; 2017f; 2021a; 2021b; 2021c). The sample size decreased as children grew older, with 10,625 families (57.3% of the original number) participating in the seventh survey (Fitzsimons, 2017).

In the first six surveys of MCS, the main respondent of a family was required to answer most of the questions about the family and the cohort member. Another member of the family, usually the partner, also completed a shorter partner/proxy survey. Priority was given to biological mothers to be the main respondents in the first survey, and the MCS team tried to keep the same individual as the main respondent in the following surveys. To improve consistency in reporting, in this study, the sample was restricted to singletons who participated in the first six surveys, and for whom the main survey respondent was the biological mother across all six surveys.

However, in the seventh survey, both parents/carers received the same questionnaire except for the Strengths and Difficulties Questionnaire (SDQ) - the tool for measuring child mental health outcomes - that was answered by one parent only. This leads to a significant reduction in the rate of SDQs answered by biological mothers compared with previous surveys. To maintain consistency in reporting the child mental health outcomes across the surveys, we further restricted the sample used in the first six surveys to those children who also participated in the seventh survey and for whom the SDQ was answered by the biological mother.

### 2.3 Variables

#### 2.3.1 Child mental health problems

The Strengths and Difficulties Questionnaire (SDQ), a validated tool for identifying behavioural and emotional problems in children (Goodman, 1997; Goodman et al., 1998; Goodman & Scott, 1999), has been included at each follow-up survey of the MCS starting when children were aged 3. The SDQ consists of 25 items grouped into five subscales: emotional problems, peer problems, conduct problems, hyperactivity and prosocial behaviour. Each subscale includes five questions, with the subscale score ranging from 0 to 10. The primary outcome in this research was mother-reported *overall child mental health problems* at ages 3, 5, 7, 11, 14 and 17 years old, measured by the Total Difficulties Score (TDS), which is calculated by adding four subscales of the SDQ (emotional, peer, and conduct problems, and hyperactivity). The score ranges from 0 to 40, with higher scores indicating worse mental health. Binary variables for TDS at different ages were generated, using validated cut-offs in which scores above 17 (16 for age 3) are categorised as ‘abnormal’ child mental health (Youthinmind, 2012) or ‘probable’ child mental health problems (Goodman et al., 2000). Continuous variables for TDS were standardised, with mean equal to 0 and standard deviation equal to 1.

The secondary outcomes were *internalising problems* (the sum of emotional problems and peer problems) and *externalising problems* (the sum of conduct problems and hyperactivity) at different ages, which both range from 0 to 20 (Goodman et al., 2010). Internalising problems include anxiety, depression and somatic complaints, whereas externalising problems include externally-focused behavioural problems such as hyperactivity, aggression and delinquent behaviour (Achenbach, 1966; Achenbach & Edelbrock, 1978). There are no validated cut-offs for internalising and externalising problems, so the scores were analysed as continuous variables after standardisation to aid direct comparison.

#### 2.3.2 Family income

In the first six surveys, the main respondents were asked to report total family income choosing from a list of pre-defined income bands. The banded income was converted into equivalised income by the MCS team. In the seventh survey, respondents were asked to report their raw family income. Due to errors in the banded income variable provided by MCS, it was necessary to derive this using the raw income data within the Secure Access data (University of London, 2021b). The raw family income was first converted into annual family income, and then equivalised using the modified OCED equivalence scale.

The primary exposure was *permanent income*, measured at each survey by averaging the equivalised annual family income up to the current survey. Income values were adjusted for inflation using the Average Weekly Earnings Index, with 2018 as the base year. The secondary exposure was *lagged transitory income*, measured by equivalised annual family income in the previous survey, which was used as a robustness check. Permanent income and transitory income were transformed into the logarithmic form.

#### 2.3.3 Covariables

Covariables were grouped as follows (Table 1): child health endowments, pregnancy-related factors, child characteristics, family socio-economic factors, parental investment variables and parental stress variables. ‘Child health endowments’ included variables that are determined before birth. Variables in the ‘pregnancy-related factors’, ‘child characteristics’ and ‘family socio-economic factors’ were treated as potential confounding factors, because they are associated with both income and child mental health problems and they are not considered to be on the causal pathway from family income to child mental health. Parental investment and parental stress variables, which may explain the mechanisms underlying the association between income and child mental health, were considered potential mediating factors. These factors, except for housing tenure, maternal psychological distress and self-reported general health, are age-specific and were collected only at certain MCS surveys. Variables details are reported in Appendix Tables A1 and A2.

**Table 1:** Variable list.

Outcomes			• Overall child mental health problems (SDQ Total Difficulties Score) • Internalising and externalising problems
Exposures			• Permanent income • Lagged transitory income
Covariables	Child health endowments		• Gestational age • Child sex • Child ethnicity • Firstborn • Child age at interview
	Potential Confounders	Pregnancy-related factors	• Maternal age at childbirth • Maternal smoking during pregnancy • Maternal alcohol consumption during pregnancy • Breastfeeding
		Child characteristics	• Child limiting physical longstanding illness • Child weight
		Family socio-economic characteristics	• Lone parent • Change in family structure • Maternal education
	Potential Mediators	Parental investment variables	• Housing tenure • Parenting activities: reads to child, teaches songs, plays sports, etc. • Home atmosphere • Childcare at 9 months and 3 years old
		Parental stress variables	• Postpartum depression • Pianta Child-Parent Relationship Scale (CPRS) at 3 years old • Maternal psychological distress in the previous survey • Maternal self-reported general health

### 2.4 Missing data

Patterns of missing data were examined within and across surveys. Inverse probability weights generated by the MCS team were used for unit-non response when a child did not participate in one or more surveys (Hansen, 2014). Multiple imputation, using the chained equation to generate 30 imputed datasets, were used to account for item non-response (Von Hippel, 2020). The imputation model included all variables that were used in the subsequent analysis.

### 2.5 Analytical approach

The analyses were conducted to investigate the relationship between family income and child mental health at 3, 5, 7, 11, 14 and 17 years of age. Conducting cross-sectional analyses can maximise the use of the rich information collected in each MCS survey by accounting for unique relevant age-specific PS and PI factors in explaining the current child mental health – family income gradient (Appendix Table A2). Multivariable logistic regression was used when TDS was analysed as a binary variable (‘abnormal’ or not), and multivariable linear regression was used when the outcomes were continuous variables (TDS, internalising and externalising problems). Marginal effects (ME) measured at the mean of all covariates were reported for logistic regression.

Model selection was conducted by forward selection (Table 2). The crude association between the exposure (family income) and the outcome (TDS, internalising and externalising problems) was first estimated. The ‘child health endowments’ variables were included on an *a priori* basis. Then variables reflecting ‘pregnancy-related factors’, ‘child characteristics’ and ‘family socio-economic factors’ were added into the models sequentially based on chronological order. The variables from the ‘Parental Stress’ and ‘Parental Investment’ theories were then added, first separately (M3 and M4 specifications) and then together (M5 specification) to explore the mechanisms underlying the child mental health-income gradient. Variables were included in the final models if they were associated with the outcome at a statistically significant 10% level or less.

**Table 2:** Model specifications.

Model	Regressors
M1	Raw correlation between family income and child mental health outcomes
M2	M1 + child health endowments, pregnancy-related factors, child characteristics and family socio-economic factors
M3	M2 + parental stress (PS) variables
M4	M2 + parental investment (PI) variables
M5	M3 + PI variables

Three sensitivity analyses were conducted to test the robustness of the results. First, we replaced permanent income with lagged transitory income. Second, we conducted a complete case analysis. Third, we repeated the cross-sectional analyses up to 14 years old using the age 17 restricted sample.

All analyses were conducted using Stata/SE 16.0 (StataCorp, 2021).

## 3 Results

### 3.1 Descriptive statistics

**Fig. 2:**
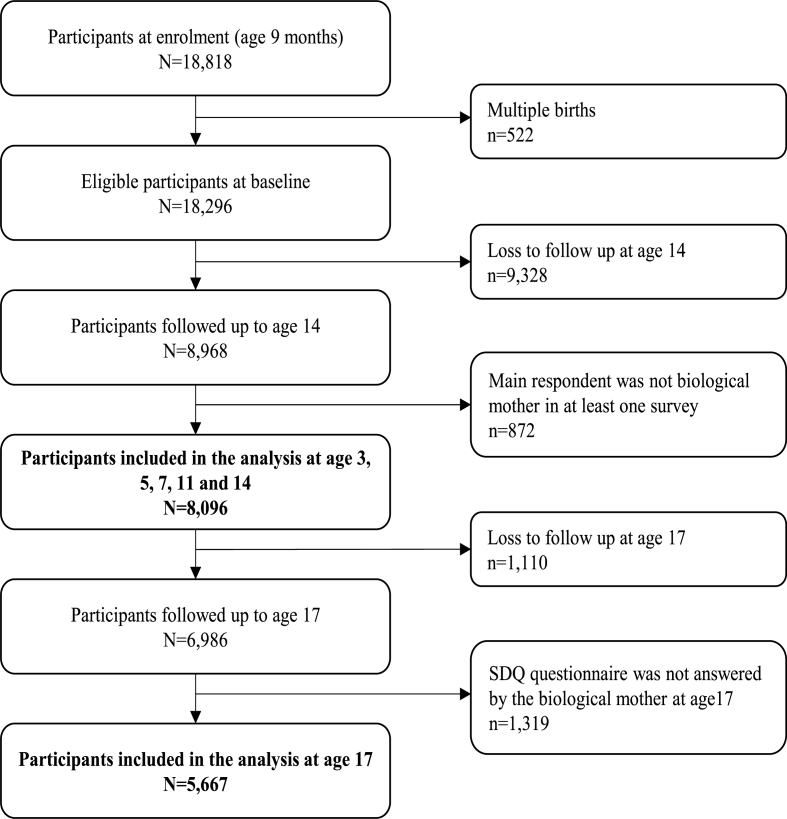
Flowchart of study participants included in the analysis. N = 8096 up to age 14; N = 5667 at age 17.

8096 singleton children were included in the analyses for ages 3 to 14 (Fig. 2). Half (50.5%) of the children were male and 87.9% were White (Table 3). The percentage of children who had TDS scores reflecting ‘abnormal’ mental health symptoms varied across surveys, from 14.5% at age 3–7.1% at 7 years, before increasing again to 10.2% at age 14. The mean internalising symptom score ranged from 2.5 to 3.8 at ages 3 to 14. It was lower in early childhood and increased as children grew older, with the highest average score of 3.8 observed at age 14. In contrast, the externalising problems score was highest at age 3 (mean score: 6.8) and decreased gradually to 4.4, on average, at age 14.

**Table 3:** Descriptive characteristics of the study population.

Variables-N (%)	3 years	5 years	7 years	11 years	14 years	17 years
	N = 8096					N = 5667
***Child mental health***						
‘Abnormal’ mental health symptoms	980 (14.5)	393 (5.9)	513 (7.3)	630 (9.6)	702 (10.2)	459 (7.9)
Total Difficulties Score-Mean (SD)	9.7 (5.2)	7.4 (5.0)	7.6 (5.4)	7.9 (5.9)	8.3 (6.1)	7.0 (5.8)
Internalising problems-Mean (SD)	2.9 (2.4)	2.5 (2.5)	2.8 (2.8)	3.3 (3.2)	3.8 (3.5)	3.7 (3.5)
Externalising problems-Mean (SD)	6.8 (3.8)	4.9 (3.5)	4.8 (3.6)	4.6 (3.6)	4.4 (3.6)	3.4 (3.2)
***Family income***						
Permanent income (£)-Mean	25,548	25,176	25,093	25,101	24,906	24,907
Permanent income (£)-SD	15,797	14,841	14,190	12,936	12,095	11,634
Lagged transitory income (£)-Mean	26,017	25,079	24,432	24,844	25,134	27,131
Lagged transitory income (£)-SD	16,982	16,742	15,083	14,605	10,536	9837
***Child health endowments***						
Age, years-Mean (SD)	3.1 (0.2)	5.2 (0.2)	7.2 (0.3)	11.2 (0.3)	14.3 (0.3)	17 (0.3)
Male	3992 (50.5)	“	“	“	“	2737 (48.8)
Minority ethnic group	1022 (12.1)	“	“	“	“	701 (8.0)
Preterm (<37 weeks gestation)	565 (7.5)	“	“	“	“	396 (7.1)
Firstborn	4064 (51.1)	“	“	“	“	2893 (51.8)
***Pregnancy-related factors***						
Maternal age at childbirth						
Less than 20 years	414 (7.9)	“	“	“	“	258 (3.7)
20–24 years	1164 (16.7)	“	“	“	“	781 (11.2)
25–29 years	2265 (28.8)	“	“	“	“	1590 (27.8)
30–34 years	2727 (29.9)	“	“	“	“	1951 (36.2)
35 or over	1526 (16.7)	“	“	“	“	1087 (21.1)
Maternal smoking during pregnancy						
Never smoked	5660 (65.3)	“	“	“	“	3991 (71.9)
Stopped smoking during pregnancy	950 (12.8)	“	“	“	“	664 (12.2)
Smoked throughout pregnancy	1486 (21.9)	“	“	“	“	1012 (15.9)
Maternal alcohol consumption during pregnancy						
Never	5462 (67.7)	“	“	“	“	3781 (62.7)
Light	2063 (25.1)	“	“	“	“	1488 (29.9)
Moderate/Heavy	571 (7.2)	“	“	“	“	398 (7.4)
Breastfeeding						
Never breastfed	2217 (33.6)	“	“	“	“	1448 (22.7)
<2 months	2145 (24.2)	“	“	“	“	1486 (25.1)
2.0–5.9 months	1640 (19.0)	“	“	“	“	1160 (21.7)
≥ 6 months	2094 (23.2)	“	“	“	“	1573 (30.5)
***Child characteristics***						
Limiting physical longstanding illness	212 (3.0)	398 (5.0)	416 (5.4)	372 (4.8)	411 (4.9)	244 (4.3)
Child weight						
Normal	6187 (77.2)	6407 (79.2)	6508 (80.3)	5888 (72.4)	5911 (72.5)	3950 (71.0)
Overweight	1455 (17.2)	1279 (15.8)	1141 (14.3)	1692 (21.2)	1585 (19.6)	1099 (19.1)
Obese	454 (5.6)	410 (5.0)	447 (5.4)	516 (6.4)	600 (7.9)	618 (9.9)
***Family socioeconomic characteristics***						
Lone parent	1057 (17.3)	1262 (20.2)	1404 (21.4)	1640 (24.8)	1750 (25.8)	1519 (26.2)
Change in family structure						
No change	7322 (88.4)	7432 (89.8)	7470 (90.1)	7195 (86.9)	7381 (89.3)	5344 (94.3)
New partner	367 (5.4)	257 (4.1)	253 (4.5)	376 (5.5)	319 (5.1)	28 (0.5)
Became single	407 (6.2)	407 (6.1)	373 (5.4)	525 (7.6)	396 (5.6)	295 (5.2)
Maternal education						
NVQ Level 1&2	2753 (38.6)	2646 (37.5)	2534 (36.1)	2367 (34.3)	2227 (32.3)	1510 (27.2)
NVQ Level 3	1234 (14.3)	1244 (14.6)	1262 (15.1)	1227 (15.0)	1204 (14.8)	837 (14.2)
NVQ Level 4&5	3196 (31.9)	3346 (33.5)	3493 (35.1)	3750 (38.1)	3965 (41.2)	2893 (53.0)
None of these	913 (15.2)	860 (14.4)	807 (13.7)	752 (12.6)	700 (11.7)	427 (5.6)

Notes: unweighted counts (N) and survey-weighted proportions (%) reported; SD standard deviation; " baseline variable which remains the same across all surveys.

Of the 8096 children used for the analyses of the first six surveys, 5667 were included in the analysis of age 17 (Fig. 2). Comparing the 3–14 years sample to the 17 years sample (Table 3), it was apparent that children's characteristics were similar in terms of sex, gestational age, and being firstborn. However, children in the restricted 17-year sample were more likely to be White and be born to older, more educated, mothers, who were also less likely to smoke during pregnancy, and more likely to breastfeed them for longer.

### 3.2 Association between family income and child mental health problems

#### 3.2.1 Overall child mental health problems (TDS)

The unadjusted association between family income and ‘abnormal’ mental health (TDS binary) was negative across all ages (Table 4), with the largest magnitude found at age 3 where a 1% increase in permanent income was associated with a decrease by 0.125 in the probability of the child presenting ‘abnormal’ mental health symptoms. After adjusting for confounding factors (Table 5), the family income marginal effects became smaller in magnitude, ranging from −0.081 (SE = 0.009) at age 11 to −0.037 (SE = 0.008) at age 7, and maintained statistically significant at all ages.

**Table 4:** Unadjusted regression analyses between permanent income and mental health problems-Model 1 crude association.

Child age	‘Abnormal’ overall	Overall	Internalising	Externalising
(years)	Logistic-Marginal effect (SE)	Linear regression-Coefficient (SE)		
3	−0.125 (0.010)	−0.506 (0.028)	−0.418 (0.027)	−0.453 (0.030)
5	−0.061 (0.005)	−0.549 (0.026)	−0.468 (0.027)	−0.483 (0.029)
7	−0.065 (0.006)	−0.522 (0.026)	−0.461 (0.027)	−0.464 (0.030)
11	−0.092 (0.008)	−0.596 (0.029)	−0.493 (0.028)	−0.564 (0.033)
14	−0.100 (0.008)	−0.668 (0.029)	−0.541 (0.029)	−0.609 (0.032)
17	−0.089 (0.007)	−0.655 (0.035)	−0.591 (0.035)	−0.535 (0.034)

Notes: All coefficients are statistically significant at the p < 0.01 level; N = 8096 up to 14; N = 5667 at age 17.

**Table 5:** Adjusted regression analyses between permanent income and mental health problems-Model 2 adjusting for confounders.

Child age	‘Abnormal’ overall	Overall	Internalising	Externalising
(years)	Logistic-Marginal effect (SE)	Linear regression-Coefficient (SE)		
3	−0.056 (0.013)	−0.228 (0.037)	−0.264 (0.036)	−0.184 (0.038)
5	−0.040 (0.005)	−0.253 (0.034)	−0.328 (0.030)	−0.163 (0.038)
7	−0.037 (0.008)	−0.311 (0.032)	−0.362 (0.032)	−0.227 (0.035)
11	−0.081 (0.009)	−0.408 (0.041)	−0.413 (0.035)	−0.332 (0.043)
14	−0.073 (0.012)	−0.476 (0.042)	−0.447 (0.038)	−0.404 (0.040)
17	−0.074 (0.008)	−0.442 (0.051)	−0.490 (0.040)	−0.313 (0.049)

Notes: All coefficients are statistically significant at the p < 0.01 level. Model 2 adjusted for child health endowments, pregnancy-related factors, child characteristics and family socio-economic factors; N = 8096 up to 14; N = 5667 at age 17.

#### 3.2.2 Internalising and externalising problems

Higher family income was strongly associated with lower internalising and externalising scores in the unadjusted model (Table 4). The magnitude of the associations was the largest at age 14 for both internalising and externalising scales, where a 1% increase in permanent income was associated with a decrease of around 0.5–0.6 of an SD in the symptom scores. Such associations decreased after adjusting for confounding factors for both internalising and externalising problems models and were consistently larger for internalising problems in all age groups (Table 5).

### 3.3 The role of Parental Stress and Parental Investment factors

#### 3.3.1 Comparison between Parental Stress and Parental Investment mechanisms

**Fig. 3:**
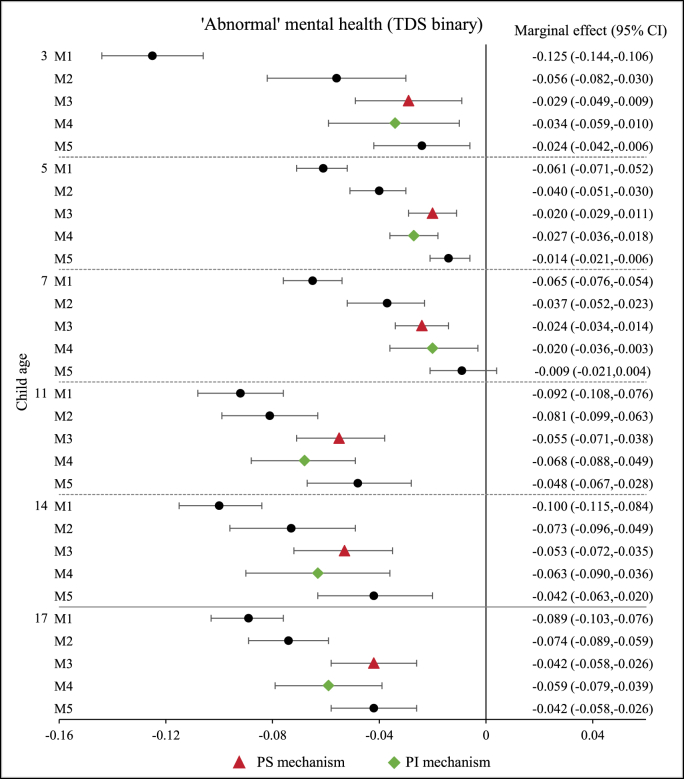
Association between family income and child ‘abnormal’ mental health. N = 8096 up to age 14; N = 5667 at age 17.

**Fig. 4:**
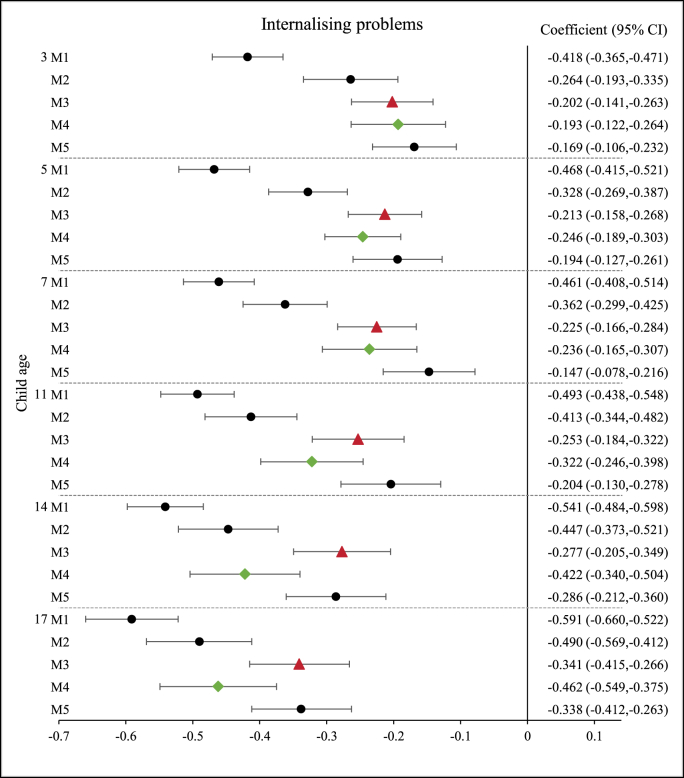
Association between family income and child internalising problems. N = 8096 up to age 14; N = 5667 at age 17.

**Fig. 5:**
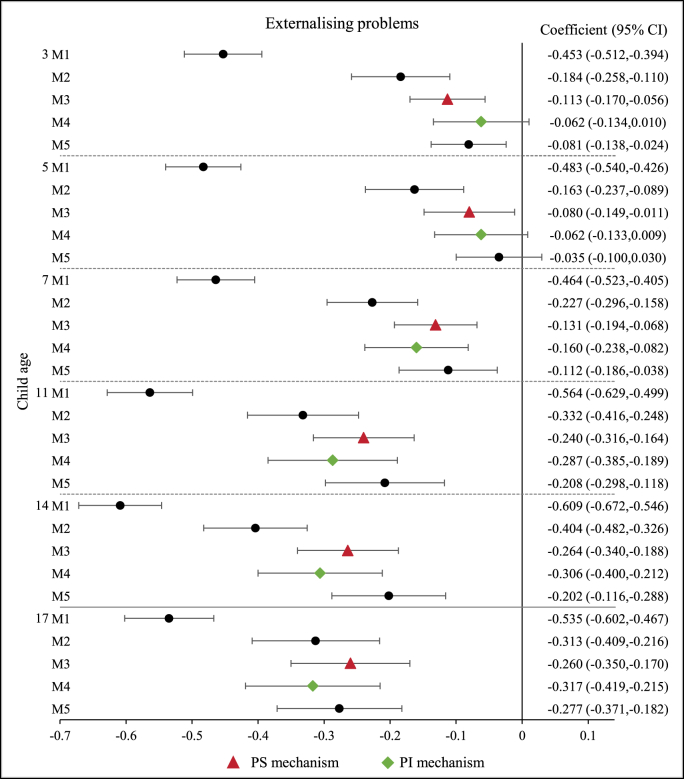
Association between family income and child externalising problems. N = 8096 up to age 14; N = 5667 at age 17.

The observed association between family income and child mental health may be explained by various underlying mechanisms. Fig. 3, Fig. 4, Fig. 5 show that, after adjustment for the PS and PI variables, the magnitude of the observed associations was reduced for all the outcomes under analysis (i.e. TDS, internalising and externalising problems scores).

For overall child mental health problems (TDS binary - Fig. 3), more of the observed marginal effect of income on ‘abnormal’ mental health was attenuated after adjusting for PS factors (Model 3) rather than PI variables (Model 4) in all age groups, except for age 7 where the opposite was true. For example, compared with the income coefficient in Model 2 (adjusted for confounding factors), the income coefficient was reduced by 27% and 14%, respectively, when the PS (Model 3) and PI (Model 4) variables were further individually added to the model at 14 years old.

For internalising and externalising problems, the relative importance of the PS and PI variables varied across age groups (Fig. 4, Fig. 5). Adjustment for PS variables reduced the association by a larger extent than adjustment for PI variables in mid-childhood and adolescence (i.e. 7, 11 and 14 years old). However, in early childhood, when children were aged 3 and 5 years, most of the observed associations between income and externalising problems were explained by the PI variables (Fig. 5). For internalising problems in early childhood, the income coefficients decreased by approximately 24% at 3 years old, when PS and PI were included separately in the model controlling for confounding factors (Fig. 4). Adjustment for PS variables yielded a larger reduction in the magnitude of the income coefficient than adjustment for PI variables at 5 years old.

#### 3.3.2 Independent association between Parental Stress and Parental Investment factors and child mental health

In the fully adjusted model (Model 5) for ‘abnormal’ child mental health (Table 6), maternal depression and child-parent relationship scale within the PS pathway, and home atmosphere within the PI pathway were consistently associated with abnormal mental health. Specifically, children whose mothers experienced depression were at increased risk of mental health problems themselves, while those whose mothers reported a good parent-child relationship and living in more organised and calm homes, were less likely to experience mental health problems. The associations between these three factors and abnormal child mental health were statistically significant in all available surveys, while there were other factors that were significant only in specific time periods, such as maternal general health within the PS pathway and time spent reading to their child within the PI pathway. Full regression models are provided in Appendix Table A3.

**Table 6:** Independent association between PS/PI and ‘abnormal’ mental health.

Variables	3 years	5 years	7 years	11 years	14 years	17 years
	N = 8096					N = 5667
Permanent income	−0.024*** (0.009)	−0.014*** (0.004)	−0.009 (0.006)	−0.048*** (0.010)	−0.042*** (0.011)	−0.042*** (0.008)
***Parental stress variables***						
Postpartum depression	0.014* (0.008)	0.010*** (0.003)	0.014*** (0.005)	0.018** (0.007)		0.012* (0.007)
CPRS at 3 years old (standardised)	−0.076*** (0.005)	−0.014*** (0.002)	−0.025*** (0.002)	−0.027*** (0.003)	−0.032*** (0.004)	−0.017*** (0.003)
Maternal psychological distress in the previous survey	NA	0.010*** (0.004)	0.022*** (0.006)	0.041*** (0.008)	0.052*** (0.008)	0.038*** (0.007)
Poor maternal general health	0.019** (0.009)		0.025*** (0.006)	0.023** (0.009)	0.028*** (0.009)	0.031*** (0.010)
***Parental investment variables***						
Childcare at 9 months						
Parental care #	–	–				
Formal care	−0.017 (0.012)	−0.009* (0.005)				
Grandparent care	−0.009 (0.012)	0.002 (0.005)				
Other informal care	−0.048*** (0.014)	−0.016*** (0.006)				
Childcare at 3 years old						
Parental care #	–					
Formal care	−0.020** (0.010)					
Grandparent care	0.012 (0.013)					
Other informal care	0.069 (0.052)					
Housing tenure						
Own/mortgaged #			–	–	–	
Private rent			0.022** (0.011)	0.016 (0.012)	0.030** (0.013)	
Social rent			0.025*** (0.008)	0.007 (0.011)	0.018 (0.013)	
Other			−0.008 (0.015)	−0.042*** (0.013)	−0.015 (0.018)	
Reads to child				NA	NA	NA
Never/Occasionally #	–	–	–			
Weekly	−0.047** (0.018)	−0.028** (0.013)	−0.027** (0.011)			
Daily	−0.057*** (0.019)	−0.036*** (0.013)	−0.026** (0.012)			
Teaches painting	NA			NA	NA	NA
Never/Occasionally #		–	–			
Weekly		−0.006 (0.004)	0.011* (0.006)			
Daily		−0.013** (0.005)	0.018 (0.016)			
Goes to parks	NA			NA	NA	NA
Never/Occasionally #			–			
Weekly			−0.009* (0.005)			
Daily			0.009 (0.016)			
Plays indoor games	NA				NA	NA
Never/Occasionally #				–		
Weekly				0.012* (0.007)		
Daily				0.002 (0.017)		
Home atmosphere	−0.007*** (0.002)	−0.004*** (0.001)	NA	NA	NA	NA

Notes: Fully adjusted Model 5 used; marginal effects (standard error) reported; # reference group; *p < 0.1 **p < 0.05 ***p < 0.01; variables that were not available in a specific survey are shown as ‘NA’; variables that did not meet model selection criteria were dropped and are not reported.

The PI and PS variables performed differently for internalising and externalising problems (Appendix Tables A4 and A5). With respect to the PI variables, parenting activities such as taking children to the library and teaching painting were strongly associated with lower externalising symptoms. However, the associations with internalising problems were weaker. For the PS factors, a better parent-child relationship was strongly associated with less internalising and externalising problems at all age groups, but the marginal effects were larger in magnitude for externalising problems. Children whose mothers experienced depression were at increased risk of internalising and externalising problems. The associations increased as children grew older, but the marginal effects were larger for internalising problems than externalising problems in magnitude in all age groups. The associations for internalising and externalising problems were statistically significant at 1% level in all age groups, except for externalising problems at age 5 where the association failed to reach statistical significance.

### 3.4 Extension to age 17

Results for children aged 17 years followed a similar pattern to those found for age 14, across all child mental health outcomes (TDS, internalising and externalising scores) both in terms of the associations with family income and in relation to the underpinning PS and PI mechanisms, and independent PS and PI associations on child mental health (Table 4, Table 5, Fig. 3, Fig. 4, Fig. 5).

### 3.5 Sensitivity analysis

#### 3.5.1 Alternative measure of exposure

The estimated income coefficients on child ‘abnormal’ mental health were smaller in magnitude using lagged transitory income than permanent income at ages 3, 5, 7, and 11 and were similar at ages 14 and 17 (Appendix Figure A1).

#### 3.5.2 Complete case analysis

Compared with the main analysis, the estimated association between family income and child mental health was smaller in magnitude for the complete case analysis for all age groups, except for age 7. The association was weaker in the complete case than the main analysis, with a statistical significance at 10% level at 3 and 5 years, and the association failed to reach statistical significance at 7 years old. (Appendix Tables A6-11).

#### 3.5.3 Restricted sample

When all analyses were conducted in the restricted sample as per age 17 (N = 5667), the estimated coefficients of family income on abnormal child mental health problems were statistically significant at least at 5% level in all model specifications at 3–14 years old (Appendix Figure A2). The coefficients were consistently smaller in magnitude in all model specifications at ages 3, 5, 11 and 14. At age 7, the estimated coefficients were smaller in the raw regression (Model 1), when adjusting for confounding factors (Model 2), and when controlling for the PS variables (Model 3) in the restricted sample.

## 4 Discussion

Using cross-sectional data from seven successive surveys of the UK Millennium Cohort Study, this study shows that family income was strongly associated with a child's mental health from early childhood to adolescence. Children who were living in low income families were more likely to have mental health problems than those living in wealthier families. After adjusting for relevant risk factors, there was a significant negative association of permanent income with probable child mental health problems at all age groups.

Our results seem to indicate that the protective effect of income on child mental health (as measured by TDS) may matter more at specific child ages. According to the life course framework, there are sensitive periods in child development where particular exposures have stronger effects (Cunha et al., 2010; Green & Popham, 2017). Early years are a crucial stage in the trajectory of child development because this is an important period for the development of cognitive functioning and social competence (Boe et al., 2017). This might explain the finding that exposure to low income has a larger association with probable mental health problems at 3 years than at 5 and 7 years of age. Another potential sensitive period for mental health problems associated with family income indicated by this study is adolescence, especially at age 11 when children have just started secondary school. This is consistent with previous findings in the UK and the US which both observed a strong and statistically significant association between long-term family income and mental health when children were aged 11 years old (Noonan et al., 2018; Votruba-Drzal, 2006). The gap in child mental health between children living in poor and rich families may be larger in early adolescence because of new pathways underlying the child mental health-income gradient, such as the growing influences of peer relationships (Jiang, 2020) and peer comparisons (Rapee et al., 2022).

When we looked at more specific mental health subtypes, we found that higher family income is associated with a lower risk of both internalising and externalising problems. Internalising problems were more strongly associated with family income than externalising problems, which is consistent with Menta (2019) using the UK Millennium Cohort Study when children were 3–14 years, and Strohschein (2005) of US children aged 4–14 years. The differences in associations between the two domains may reflect internalising problems (e.g. depression, anxiety) being more responsive to long-term family income than externalising problems (e.g. conduct problems). This corroborates findings from Fletcher and Wolfe (2016) who used permanent income to investigate the effect of family income on child externalising and internalising problems for US children aged 6–12 years. Fletcher and Wolfe (2016) suggests a negative income gradient on externalising and internalising problems at 6 years old; the gradient on externalising problems was relatively constant, while the gradient on internalising problems increased when children grew older.

The presence of a statistically significant protective effect of family income on child mental health assessed at different points during childhood and early adolescence suggests that reducing child poverty by income transfers might be a way to reduce child mental health inequalities (Noonan et al., 2018). Unconditional household income transfer has been found to improve adolescents' mental health in a quasi-experimental study in the US, and the impact was especially significant for adolescents experiencing more severe mental health problems in their childhood (Akee et al., 2018). Child benefit programs, namely conditional cash transfer programs targeting eligible families to help with the cost of bringing up children, currently exist in a number of countries such as Australia, the UK, and Ireland. Research showed that increased child benefits improved children's mental health and test scores in Canada (Milligan & Stabile, 2011). The reduction of family income in already disadvantaged families may increase child poverty and further broaden the gap of income-related child mental health inequalities.

While there was a large unadjusted association between family income and child mental health in our study, much of this association was attenuated by other risk factors such as variables derived from Parental Investment and Parental Stress theory. Consequently, the fully-adjusted association was relatively small. This suggests that focusing on family income may have broader benefits to the wider family, reducing the prevalence of other income-patterned risk factors (Cooper & Stewart, 2020). Moreover, the results suggest that Parental Stress variables may have a larger role than Parental Investment variables in explaining the relationship between income and child mental health. This is consistent with previous studies using earlier MCS surveys of children aged 3 and 5 years (Violato et al., 2011), and Australian data when children were aged 4–13 years (Khanam & Nghiem, 2016). The larger association with Parental Stress may be explained by the specific mechanisms of the Parental Investment and Parental Stress frameworks. Parental investment mechanisms are hypothesised to work through the impact of cognitive ability on child development (Sosu & Schmidt, 2017). It is hypothesised that lower family income restricts parents' ability to invest in better educational experiences for children, which in turn may have a negative effect on children's cognitive ability. Therefore, parental investment may be more important for a child's cognitive development than for non-cognitive outcomes such as emotional problems (Yeung et al., 2002). When it comes to the Parental Stress mechanisms, previous research has found that lower family income has an impact on parental stress that increases the use of harsh discipline, which directly affects a child's mental health (Sosu & Schmidt, 2017). Important Parental Stress factors such as maternal distress have also been shown to have a direct and strong impact on children's mental health (Halligan et al., 2007; Trapolini et al., 2007).

With respect to the independent association between specific risk factors and children's mental health, the results are consistent with previous studies that have highlighted an important role of maternal depression in both childhood and adolescence. Previous research found that postpartum depression was a significant risk factor for children's mental health and attenuated the child mental health-income gradient at age 5 (Rutherford et al., 2019). Our study provides evidence that the associations with recent maternal depression were stronger than associations between postpartum depression and poor child mental health from 5 to 17 years old, especially for children's internalising problems. While the independent association between risk factors in early childhood (e.g. mother-child relationship quality at age 3, early years childcare, postpartum depression) and current child mental health problems became smaller when children grew up, the association between more recent maternal psychological distress and child mental health problems increased. It is possible that a recent risk or protective factor plays a more important role in child mental health than the factors in the early years. This finding might also be explained by the reduction of reporting bias over time, as respondents were more familiar with the rating process and the criteria they should consider.

Sensitivity analyses showed that lagged transitory income had a smaller association with child mental health than permanent income in most cases, which is in line with previous studies (Blau, 1999; Violato et al., 2011). This suggests that consistent exposure to low income rather than experiencing the occasional periods of low income is more harmful to child mental health. A smaller association was also observed in the complete case and when using a restricted sample with fewer participants than the main analysis. This may indicate a selection bias, where children whose mothers completed all relevant questions and participated in all seven surveys had better mental health and lived in higher-income families compared to those with missing responses or who dropped out of the surveys.

### 4.1 Strengths and limitations of the study

Strengths of the study include the comprehensive analysis of the relationship between family income and child mental health using consistent cohorts in the seven cross-sectional surveys of the MCS, which include repeated measures of income and validated measures of mental health. Most of the previously published cross-sectional studies have focused on a specific age group (Collishaw et al., 2019; Kiernan & Mensah, 2009; Lai et al., 2019), which makes it difficult to compare the change in income gradient as a child ages. This study provides evidence for the same group of children from early childhood to adolescence. Restricting the sample to children for which the person completing the survey was always the biological mother further improved consistency. Using measures of total difficulties, internalising, and externalising outcomes has enabled us to compare the association between family income and both overall and distinct aspects of child mental health problems, which may have differential implications for policy and practice for children's mental health. Moreover, the use of permanent income in this study helped to address the limited evidence on the impact of long-term family income on children's mental health at different developmental ages. Finally, although some studies have used longitudinal analyses to investigate the trajectory of family income on child mental health (Garratt et al., 2017; Menta, 2019; Schenck-Fontaine & Panico, 2019), they did not examine the effects of age-specific factors underlying the association. These factors, such as parenting activities, being age-specific, evolve as the child grows, and their specific association with the current child mental health is best captured in age-specific cross-sectional analyses.

The study also has some limitations that should be considered when interpreting the results. Our findings do not allow for a causal interpretation. Despite controlling for a rich set of variables, there may be still unobserved heterogeneity which may bias our estimates. Linked to the above issue, problems of reverse causality may arise if child mental health impacts family income and other factors. We reduced the potential bias by averaging family income in all available surveys and by using lagged transitory family income in the sensitivity analyses. The bias from the important risk factor ‘maternal depression’ was reduced using the variable collected in the previous survey. However, the problem of reverse causality from other factors, such as parenting activities, cannot be eliminated. Future research may address these issues in more depth by applying panel data and instrumental variables methods (Wooldridge, 2015) or cross-lagged panel models (Hamaker et al., 2015; Mulder & Hamaker, 2021).

Another limitation is the amount of missing data, which is unavoidable in longitudinal studies (descriptive statistics of missing data see Appendix Table A12). As indicated from the sensitivity analysis, conducting a complete case analysis excluded participants from disadvantaged backgrounds (Appendix Table A13), which underestimated the association between family income and child mental health. We used attrition weights to address any differential loss to follow up at age 17 and multiple imputation for item-non response. However, our study population was also restricted to children where the biological mother had answered the SDQ at each survey (around 90% of all children up to age 14 and 80% at age 17), which may have reduced generalisability. For example, the percentage of the ethnic minority group in this study ranged from 8.0% to 12.1% of the study population (Table 3), which was lower compared to the original MCS sample with a percentage of 13.5% (Fitzsimons, 2020).

Finally, there might be reporting bias in measures of child mental health problems. Recent published research using the MCS data has shown that parent-reported SDQ measures satisfy longitudinal measurement invariance from 5 to 14 years old, i.e. measurement is comparable across different developmental stages, but not at 3 and 17 years old (Murray et al., 2022). Consistently, our study observed a relatively high percentage of probable mental health problems at 3 years compared with other age groups (Table 3), which was mainly driven by the high prevalence of conduct problems at this age, 18.5% and 16.3% for boys and girls, respectively. Some behaviours, such as aggressive behaviours, may be captured as conduct problems in the SDQ, while they may be common and normal behaviours in preschool children (Nærde et al., 2014). The high proportion of children with probable mental health problems at age 3 may therefore indicate that child mental health symptoms are over-identified, which may then, in turn, bias the estimates of the association between income and child mental health.

## 5 Conclusions

This study found that lower family income is associated with worse child mental health in childhood and adolescence, and much of the observed association is attenuated by other factors such as maternal depression. This suggests that focusing policy on family income may have benefits both in terms of child mental health but also broader benefits to the wider family, reducing the prevalence of income-patterned risk factors. Evidence from this study may help inform policies to support family income in disadvantaged families, which ultimately may contribute to the reduction of child mental health inequalities.

## Ethical statement

The research ‘Child mental health and income gradient from early childhood to adolescence: Evidence from the UK’ is a secondary analysis of publicly available, de-identified data drawn from the UK Millennium Cohort Study. The study was originally approved by Multi-Centre Research Ethics Committee (Shepherd & Gilbert, 2019) and this analysis did not require further ethical approvals. Individual level income data was available for analysis via the UK Data Archive's Secure Data Service, after approval by the UK Data Service (study ref: SN8753).

## Author statement

**Murong Yang**: Conceptualization, Methodology, Formal analysis, Writing - Original draft preparation **Claire Carson**: Conceptualization, Methodology, Writing - Review & Editing, Supervision **Cathy Creswell**: Writing - Review & Editing **Mara Violato**: Conceptualization, Methodology, Writing - Review & Editing, Supervision.

## Declaration of competing interest

The authors declare that they have no known competing financial interests or personal relationships that could have appeared to influence the work reported in this paper.

## Data Availability

The data is public available and can be downloaded from https://cls.ucl.ac.uk/cls-studies/millennium-cohort-study/. The code will be made available on request.
