# Supplementary material for: Child mental health and income gradient from early childhood to adolescence: Evidence from the UK
Source: SSM Popul Health. 2023 Oct 14;24:101534. doi: 10.1016/j.ssmph.2023.101534 (PMC10638036; doi:10.1016/j.ssmph.2023.101534)
Supplement: Multimedia component 1 [file mmc1.docx]

# Appendix

## Table A1. Covariable list

| Variable | Type | Category | Description |
| --- | --- | --- | --- |
| **Child health endowment** | | | |
| Child age at interview | Continuous | _ | _ |
| Child sex | Binary | 0=Female; 1=Male | _ |
| Child ethnicity | Binary | 0=White; 1= Minority ethnic group | _ |
| Gestational age | Binary | 0=Term (37-41 weeks); 1=Pre-term (<37 weeks) | Excluded post-term infants (42-43 weeks) based on Boyle *et al*.(2012) [1] |
| Firstborn | Binary | 0=Not firstborn 1=firstborn | _ |
| **Potential confounding factors** | | | |
| **Pregnancy-related factors** | | | |
| Maternal age at childbirth | Categorical | Less than 20 years; 20-24 years; 25-29 years; 30-34 years; 35 years or over | _ |
| Maternal smoking during pregnancy | Categorical | Never smoked; stopped smoking during pregnancy; smoked throughout pregnancy | _ |
| Maternal alcohol consumption during pregnancy | Categorical | Never; light; moderate/heavy | _ |
| Breastfeeding | Categorical | Never breastfed; <2 months; 2.0-5.9 months; >=6 months | _ |
| **Child characteristics** | | | |
| Child limiting longstanding illness | Binary | 0=No illness; 1=Had illness | Illness coded by ICD10; excluded mental and behavioural disorders. |
| Child weight | Categorical | Normal; overweight; obese | Comparing cohort member’s Body Mass Index (BMI) with the International Obesity TaskForce (IOTF) thresholds for overweight and obesity. |
| **Family socio-economic characteristics** | | | |
| Lone parent | Binary | 0= two-parent family; 1=lone-parent family | _ |
| Change in family structure | Categorical | No change; New partner; became single | _ |
| Maternal education | Categorical | NVQ Level 1&2; NVQ Level 3; NVQ Level 4&5; none of these | NVQ stands for national vocational qualification; a higher NVQ level indicates a higher educational level. |
| **Potential mediating factors** | | | |
| **Parental stress variables** | | | |
| Postpartum depression | Binary | 0=No depression; 1=Depression | This variable was generated by combining two variables: postpartum depression and Malaise Inventory. Postpartum depression was derived from a question ‘Since ^Jack was born, has there ever been a time lasting two weeks or more when you felt low or sad?’. The answer was ‘Yes’ or ‘No’. Malaise Inventory Score consisted of 9 questions related to depression in the MCS. A question will be coded as 1 if the answer is ‘Yes’ and 0 otherwise. A score of 4 or higher indicating that the respondent was experiencing depression. It captured maternal depression when the child was at 9 months. To fully capture postpartum depression in this thesis, the two variables were combined, where the mother was treated as experiencing postpartum depression if she satisfied either of the two conditions. |
| Child-parent relationship scale (CPRS) | Continuous | _ | CPRS assesses the relationship between parents and the child from parent’ view. It consists of 15 items with a scale of 1-5 for each item [2]. In this thesis, the higher the CPRS score, the better the relationship between the mother and the child. Full questionnaire could be found in Appendix. |
| Maternal psychological distress | Binary | 0=No distress; 1=Distress | This variable was generated by combing two variables: Kessler Psychological Distress Scale (K6+) and ‘whether treated for depression’. Kessler 6 is a self-reported measure of psychological distress. It consists of 6 items, with a scale of 0-4 for each item. A total score of 8 or higher indicating symptoms of psychological distress [3]. Full questionnaire could be found in Appendix. The second variable was derived from a question ‘Can I check, are you currently being treated for depression or serious anxiety?’. The answer was ‘Yes’ or ‘No’. To fully capture maternal distress in this thesis, the two variables were combined, where the mother was treated as experiencing psychological distress if she satisfied either of the two conditions. The lagged variable was used in this thesis to reduce reverse causality, i.e. assessment of a child’s mental health status may be biased if the mother had mental health problems. |
| Mother’s self-reported general health | Binary | 0=Good; 1=Poor | This variable was derived from a question asking the respondent to describe her health generally. |
| **Parental investment variables** | | | |
| Housing tenure | Categorical | Own/mortgaged; private rent; social rent; others | _ |
| Parenting activities: reads to child, teaches songs, plays sports, etc. | Categorical | Never/occasionally; weekly; daily | _ |
| Home atmosphere | Continuous | _ | This measures how much calmness and how organised a home is. The higher the score, the better the home atmosphere. Full questionnaire could be found in Appendix. |
| Childcare at 9 months and 3 years old | Categorical | Parent care; formal childcare; grandparent care; other informal care | Childcare was categories: as ‘formal’ if the care was provided by nanny, au pair, childminder or nursery; or ‘other informal care’ if the care was provided by other relative, a friend or neighbour. |

|  | | Variable available in the survey | | | | | Yes | No | Variable used in the cross-sectional analysis | | | | | |
| --- | --- | --- | --- | --- | --- | --- | --- | --- | --- | --- | --- | --- | --- | --- |
| Child age (year) | | 9 months | 3 | 5 | 7 | 11 | 14 | 17 | 3 | 5 | 7 | 11 | 14 | 17 |
| PS | Postpartum depression |  |  |  |  |  |  |  |  |  |  |  |  |  |
|  | Child-Parent Relationship Scale (CPRS) |  |  |  |  |  |  |  |  |  |  |  |  |  |
|  | Maternal distress in the previous sweep |  |  |  |  |  |  |  |  |  |  |  |  |  |
|  | Mother’s self-reported general health |  |  |  |  |  |  |  |  |  |  |  |  |  |
| PI | Housing tenure |  |  |  |  |  |  |  |  |  |  |  |  |  |
|  | Home atmosphere |  |  |  |  |  |  |  |  |  |  |  |  |  |
|  | Childcare at 9 months |  |  |  |  |  |  |  |  |  |  |  |  |  |
|  | Childcare at 3 years old |  |  |  |  |  |  |  |  |  |  |  |  |  |
|  | Takes child to the library |  |  |  |  |  |  |  |  |  |  |  |  |  |
|  | Helps child learn alphabet |  |  |  |  |  |  |  |  |  |  |  |  |  |
|  | Teaches counting |  |  |  |  |  |  |  |  |  |  |  |  |  |
|  | Teaches songs/poems/nursery rhymes |  |  |  |  |  |  |  |  |  |  |  |  |  |
|  | Reads to child |  |  |  |  |  |  |  |  |  |  |  |  |  |
|  | Does musical activities |  |  |  |  |  |  |  |  |  |  |  |  |  |
|  | Paints, draws or makes things |  |  |  |  |  |  |  |  |  |  |  |  |  |
|  | Goes to parks or playgrounds |  |  |  |  |  |  |  |  |  |  |  |  |  |
|  | Plays active games |  |  |  |  |  |  |  |  |  |  |  |  |  |
|  | Plays indoor games |  |  |  |  |  |  |  |  |  |  |  |  |  |
|  | Tells stories |  |  |  |  |  |  |  |  |  |  |  |  |  |
|  | Talks things important to child |  |  |  |  |  |  |  |  |  |  |  |  |  |

## Table A2. List of parental stress and parental investment variables in the study

## Table A3. Independent associations between risk/protective factors and ‘abnormal’ child mental health– Fully adjusted Model 5

|  | 3 years | 5 years | 7 years | 11 years | 14 years | 17 years |
| --- | --- | --- | --- | --- | --- | --- |
| **Variables** |  | N=8096 |  |  |  | N=5667 |
| Permanent income | -0.024***(0.009) | -0.014***(0.004) | -0.009(0.006) | -0.048***(0.010) | -0.042***(0.011) | -0.042***(0.008) |
| **Child health endowments** |  |  |  |  |  |  |
| Child age | -0.029(0.021) | -0.001(0.007) | -0.003(0.009) | -0.022**(0.010) | -0.010(0.012) | -0.003(0.010) |
| Male child | 0.016**(0.007) | 0.013***(0.004) | 0.026***(0.005) | 0.036***(0.007) | 0.025***(0.007) | 0.011(0.007) |
| Minority ethnic group | 0.046***(0.012) | 0.004(0.005) | 0.001(0.007) | -0.030**(0.014) | -0.023***(0.011) | -0.014(0.010) |
| Pre-term | 0.011(0.014) | <0.001(0.006) | -0.002(0.010) | 0.010(0.011) | 0.015(0.012) | 0.006(0.012) |
| Firstborn | 0.008(0.009) | 0.008**(0.004) | 0.007(0.005) | 0.013*(0.007) | -0.006(0.008) | -0.003(0.007) |
| **Pregnancy-related factors** |  |  |  |  |  |  |
| Maternal age at childbirth |  |  |  |  |  |  |
| Less than 20 years | 0.022(0.020) | 0.005(0.007) |  |  |  |  |
| 20 to 24 years | 0.036***(0.014) | 0.012**(0.005) |  |  |  |  |
| 25 to 29 years # | _ | _ |  |  |  |  |
| 30 to 34 years | -0.008(0.008) | 0.003(0.004) |  |  |  |  |
| 35 or over | -0.003(0.009) | 0.006(0.006) |  |  |  |  |
| Maternal smoking during pregnancy |  |  |  |  |  |  |
| Never smoked # |  |  |  |  |  |  |
| Stopped smoking during pregnancy |  |  |  |  |  |  |
| Smoked throughout pregnancy |  |  |  |  |  |  |
| Maternal alcohol consumption during pregnancy |  |  |  |  |  |  |
| Never # |  |  |  | _ | _ |  |
| Light |  |  |  | -0.019**(0.008) | -0.003(0.009) |  |
| Moderate/Heavy |  |  |  | 0.016(0.013) | 0.034**(0.016) |  |
| Breastfeeding |  |  |  |  |  |  |
| Never breastfed # | _ | _ | _ | _ | _ |  |
| <2 months | -0.030***(0.011) | -0.001(0.004) | 0.004(0.007) | 0.021**(0.008) | 0.024***(0.009) |  |
| 2.0-5.9 months | -0.055***(0.013) | <-0.001(0.006) | -0.012*(0.007) | -0.003(0.011) | 0.002(0.011) |  |
| ≥ 6 months | -0.056***(0.012) | -0.010**(0.004) | -0.020***(0.006) | -0.011(0.009) | -0.003(0.010) |  |
| **Child characteristics** |  |  |  |  |  |  |
| Limiting longstanding illness | 0.060***(0.018) | 0.013*(0.007) | 0.025**(0.010) | 0.020*(0.012) |  |  |
| Child weight |  |  |  |  |  |  |
| Normal # |  |  |  | _ | _ | _ |
| Overweight |  |  |  | 0.017*(0.010) | 0.025**(0.010) | 0.009(0.008) |
| Obese |  |  |  | 0.016(0.014) | 0.036**(0.016) | 0.043***(0.014) |
| **Family socio-economic characteristics** | |  |  |  |  |  |
| Lone parent |  |  |  |  |  | 0.020***(0.008) |
| Change in family structure |  |  |  |  |  | _ |
| No change # |  |  |  |  |  | -0.043***(0.011) |
| New partner |  |  |  |  |  | -0.003(0.012) |
| Became single |  |  |  |  |  |  |
| Maternal education |  |  |  |  |  |  |
| NVQ Level 1&2 # |  | _ |  |  |  |  |
| NVQ Level 3 |  | 0.005(0.005) |  |  |  |  |
| NVQ Level 4&5 |  | <-0.001(0.004) |  |  |  |  |
| None of these |  | 0.011*(0.006) |  |  |  |  |
| **Mediating factors – Parental Stress** | |  |  |  |  |  |
| Postpartum depression | 0.014*(0.008) | 0.010***(0.003) | 0.014***(0.005) | 0.018**(0.007) |  | 0.012*(0.007) |
| CPRS (standardised) | -0.076***(0.005) | -0.014***(0.002) | -0.025***(0.002) | -0.027***(0.003) | -0.032***(0.004) | -0.017***(0.003) |
| Maternal psychological distress in the previous survey | NA | 0.010***(0.004) | 0.022***(0.006) | 0.041***(0.008) | 0.052***(0.008) | 0.038***(0.007) |
| Maternal poor general health | -0.019**(0.009) |  | -0.025***(0.006) | -0.023**(0.009) | -0.028***(0.009) | 0.031***(0.010) |
| **Mediating factors – Parental Investment** | |  |  |  |  |  |
| Childcare at 9 months |  |  |  |  |  |  |
| Parental care # | - | - |  |  |  |  |
| Formal care | -0.017(0.012) | -0.009*(0.005) |  |  |  |  |
| Grandparent care | -0.009(0.012) | 0.002(0.005) |  |  |  |  |
| Other informal care | -0.048***(0.014) | -0.016***(0.006) |  |  |  |  |
| Childcare at 3 years old |  |  |  |  |  |  |
| Parental care # | - |  |  |  |  |  |
| Formal care | -0.020**(0.010) |  |  |  |  |  |
| Grandparent care | 0.012(0.013) |  |  |  |  |  |
| Other informal care | 0.069(0.052) |  |  |  |  |  |
| Housing tenure |  |  |  |  |  |  |
| Own/mortgaged # |  |  | - | - | - |  |
| Private rent |  |  | 0.022**(0.011) | 0.016(0.012) | 0.030**(0.013) |  |
| Social rent |  |  | 0.025***(0.008) | 0.007(0.011) | 0.018(0.013) |  |
| Other |  |  | -0.008(0.015) | -0.042***(0.013) | -0.015(0.018) |  |
| Reads to child |  |  |  | NA | NA | NA |
| Never/Occasionally # | - | - | - |  |  |  |
| Weekly | -0.047**(0.018) | -0.028**(0.013) | -0.027**(0.011) |  |  |  |
| Daily | -0.057***(0.019) | -0.036***(0.013) | -0.026**(0.012) |  |  |  |
| Teaches painting | NA |  |  | NA | NA | NA |
| Never/Occasionally # |  | - | - |  |  |  |
| Weekly |  | -0.006(0.004) | 0.011*(0.006) |  |  |  |
| Daily |  | -0.013**(0.005) | 0.018(0.016) |  |  |  |
| Goes to parks | NA |  |  | NA | NA | NA |
| Never/Occasionally # |  |  | - |  |  |  |
| Weekly |  |  | NA |  |  |  |
| Daily |  |  | 0.009(0.016) |  |  |  |
| Plays indoor games | NA |  |  |  | NA | NA |
| Never/Occasionally # |  |  |  | - |  |  |
| Weekly |  |  |  | 0.012*(0.007) |  |  |
| Daily |  |  |  | 0.002(0.017) |  |  |
| Home atmosphere | -0.007***(0.002) | -0.004***(0.001) | NA | NA | NA | NA |

Notes: marginal effects (standard error) reported; # reference group; * p<0.1 ** p<0.05 *** p<0.01; variables that were not available in a specific survey are shown as ‘NA’; variables that did not meet model selection criteria were dropped and are not reported.

## Table A4. Independent associations between risk/protective factors and internalising problems – Fully adjusted Model 5

|  | 3 years | 5 years | 7 years | 11 years | 14 years | 17 years |
| --- | --- | --- | --- | --- | --- | --- |
| **Variables** |  | N=8096 |  |  |  | N=5667 |
| Permanent income | -0.169***(0.032) | -0.194***(0.034) | -0.147***(0.035) | -0.204***(0.038) | -0.286***(0.038) | -0.338***(0.038) |
| **Child health endowments** |  |  |  |  |  |  |
| Child age | -0.107(0.076) | -0.101*(0.061) | -0.068(0.051) | -0.111***(0.043) | -0.081**(0.041) | -0.105***(0.039) |
| Male child | 0.059**(0.024) | 0.019(0.028) | 0.040(0.027) | 0.011(0.030) | -0.121***(0.028) | -0.227***(0.028) |
| Minority ethnic group | 0.332***(0.052) | 0.262***(0.051) | 0.160***(0.045) | -0.029(0.046) | -0.096**(0.047) | -0.151***(0.048) |
| Pre-term | 0.109*(0.061) | 0.123**(0.056) | 0.034(0.061) | 0.059(0.056) | 0.150**(0.060) | 0.074(0.064) |
| Firstborn | 0.109***(0.028) | 0.134***(0.029) | 0.164***(0.028) | 0.112***(0.028) | 0.071**(0.028) | 0.073***(0.028) |
| **Pregnancy-related factors** |  |  |  |  |  |  |
| Breastfeeding |  |  |  |  |  |  |
| Never breastfed # | - |  |  | - | - |  |
| <2 months | -0.099***(0.035) |  |  | 0.097**(0.038) | 0.118***(0.037) |  |
| 2.0-5.9 months | -0.169***(0.045) |  |  | 0.043(0.040) | 0.056(0.038) |  |
| ≥ 6 months | -0.136***(0.037) |  |  | 0.060*(0.036) | 0.045(0.034) |  |
| **Child characteristics** |  |  |  |  |  |  |
| Limiting longstanding illness | 0.408***(0.101) | 0.261***(0.072) | 0.334***(0.067) | 0.474***(0.074) | 0.178***(0.062) | 0.211***(0.089) |
| Child weight |  |  |  |  |  |  |
| Normal # | - | - | - | - | - | _ |
| Overweight | -0.073**(0.031) | -0.039(0.036) | 0.060(0.038) | 0.146***(0.040) | 0.118***(0.039) | 0.105***(0.039) |
| Obese | 0.043(0.061) | 0.160***(0.061) | 0.211***(0.072) | 0.322***(0.064) | 0.360***(0.071) | 0.266***(0.057) |
| **Family socio-economic characteristics** | | | | | | |
| Lone parent | -0.124**(0.048) |  |  |  | 0.083**(0.040) | 0.125***(0.044) |
| Maternal education |  |  |  |  |  |  |
| NVQ Level 1&2 # | - | - | - | - | - | - |
| NVQ Level 3 | -0.051(0.033) | 0.040(0.039) | -0.045(0.038) | -0.002(0.041) | -0.064(0.039) | -0.423***(0.116) |
| NVQ Level 4&5 | -0.090***(0.027) | -0.024(0.028) | -0.050*(0.027) | -0.078**(0.035) | -0.103***(0.035) | -0.062(0.075) |
| None of these | 0.081(0.056) | 0.209***(0.056) | 0.067(0.053) | 0.108*(0.060) | -0.037(0.063) |  |
| **Mediating factors – Parental Stress** | | | | | | |
| Postpartum depression | 0.107***(0.031) | 0.124***(0.032) | 0.140***(0.034) | 0.148***(0.038) | 0.124***(0.033) | 0.120***(0.035) |
| CPRS (standardised) | -0.271***(0.014) | -0.193***(0.016) | -0.206***(0.017) | -0.163***(0.016) | -0.161***(0.015) | -0.137***(0.016) |
| Maternal psychological distress in the previous survey | NA | 0.149***(0.047) | 0.257***(0.052) | 0.264***(0.048) | 0.312***(0.041) | 0.353***(0.042) |
| Poor maternal general health | -0.142***(0.045) | -0.219***(0.058) | -0.289***(0.051) | -0.352***(0.057) | -0.254***(0.045) | 0.279***(0.062) |
| **Mediating factors - Parental Investment** | | | | | | |
| Childcare at 9 months |  |  |  |  |  |  |
| Parental care # | - |  | - |  |  |  |
| Formal care | -0.063*(0.038) |  | -0.123***(0.036) |  |  |  |
| Grandparent care | -0.029(0.047) |  | 0.004(0.038) |  |  |  |
| Other informal care | -0.173**(0.079) |  | -0.193***(0.069) |  |  |  |
| Childcare at 3 years old |  |  |  |  |  |  |
| Parental care # | - | - |  |  | - |  |
| Formal care | -0.054(0.041) | -0.009(0.035) |  |  | 0.033(0.038) |  |
| Grandparent care | 0.124***(0.046) | 0.112***(0.037) |  |  | 0.077**(0.037) |  |
| Other informal care | 0.045(0.114) | 0.035(0.109) |  |  | 0.249***(0.082) |  |
| Housing tenure |  |  |  |  |  |  |
| Own/mortgaged # |  | - | - | - |  |  |
| Private rent |  | -0.119**(0.055) | 0.019(0.051) | 0.050(0.048) |  |  |
| Social rent |  | 0.025(0.050) | 0.130**(0.052) | 0.089(0.055) |  |  |
| Other |  | -0.079(0.085) | -0.014(0.096) | -0.181*(0.097) |  |  |
| Reads to child |  |  |  | NA | NA | NA |
| Never/Occasionally # | - | - | - |  |  |  |
| Weekly | -0.155**(0.067) | -0.144*(0.078) | -0.103**(0.051) |  |  |  |
| Daily | -0.155**(0.069) | -0.141*(0.077) | -0.069(0.054) |  |  |  |
| Plays active games | NA |  | Dropped |  | NA | NA |
| Never/Occasionally |  | - |  | - |  |  |
| Weekly |  | -0.051*(0.029) |  | -0.032(0.031) |  |  |
| Daily |  | 0.066(0.066) |  | 0.203*(0.104) |  |  |
| Plays indoor games | NA |  |  |  | NA | NA |
| Never/Occasionally # |  |  |  | - |  |  |
| Weekly |  |  |  | 0.068**(0.030) |  |  |
| Daily |  |  |  | 0.091(0.085) |  |  |
| Talks things important to child | NA | NA | NA | NA | NA |  |
| Never/Occasionally |  |  |  |  |  | - |
| Weekly |  |  |  |  |  | -0.191**(0.097) |
| Daily |  |  |  |  |  | -0.231**(0.096) |
| Home atmosphere | -0.015**(0.007) | -0.053***(0.007) | NA | NA | NA | NA |

Notes: marginal effects (standard error) reported; # reference group; * p<0.1 ** p<0.05 *** p<0.01; variables that were not available in a specific survey are shown as ‘NA’; variables that did not meet model selection criteria were dropped and are not reported.

## Table A5. Independent association between risk/protective factors and externalising problems – Fully adjusted Model 5

|  | 3 years | 5 years | 7 years | 11 years | 14 years | 17 years |
| --- | --- | --- | --- | --- | --- | --- |
| **Variables** | N=8096 |  |  |  |  | N=5667 |
| Permanent income | -0.081***(0.029) | -0.035(0.033) | -0.112***(0.038) | -0.208***(0.046) | -0.207***(0.046) | -0.277**(0.048)* |
| **Child health endowments** |  |  |  |  |  |  |
| Child age | -0.229***(0.068) | -0.164***(0.052) | -0.136**(0.056) | -0.141***(0.044) | -0.041(0.040) | -0.043(0.039) |
| Male child | 0.136***(0.026) | 0.244***(0.026) | 0.324***(0.026) | 0.334***(0.028) | 0.280***(0.030) | 0.221***(0.025) |
| Minority ethnic group | 0.043(0.043) | 0.104**(0.047) | -0.109**(0.055) | -0.190***(0.047) | -0.085*(0.046) | -0.057(0.044) |
| Pre-term | -0.005(0.046) | 0.039(0.049) | 0.071(0.049) | 0.093*(0.051) | 0.102*(0.057) | 0.040(0.057) |
| Firstborn | -0.035(0.025) | 0.007(0.029) | 0.006(0.030) | -0.031(0.027) | -0.067**(0.030) | -0.001(0.031) |
| **Pregnancy-related factors** |  |  |  |  |  |  |
| Maternal age at childbirth |  |  |  |  |  |  |
| Less than 20 years | 0.205***(0.067) | 0.117(0.078) | 0.057(0.082) | 0.081(0.075) | 0.061(0.080) | 0.070(0.080) |
| 20 to 24 years | 0.221***(0.041) | 0.195***(0.045) | 0.167***(0.044) | 0.143***(0.049) | 0.173***(0.051) | 0.169***(0.058) |
| 25 to 29 years # | - | - | - | - | - | _ |
| 30 to 34 years | -0.031(0.026) | 0.003(0.034) | -0.061*(0.034) | -0.043(0.033) | -0.047(0.033) | -0.036(0.038) |
| 35 or over | -0.020(0.030) | -0.025(0.041) | -0.053(0.038) | -0.061*(0.037) | -0.071*(0.039) | -0.007(0.042) |
| Maternal smoking during pregnancy |  |  |  |  |  |  |
| Never smoked # |  |  | - | - | - | _ |
| Stopped smoking during pregnancy |  |  | 0.009(0.040) | 0.001(0.038) | 0.015(0.040) | 0.048(0.045) |
| Smoked throughout pregnancy |  |  | 0.098**(0.043) | 0.111***(0.042) | 0.145***(0.045) | 0.125***(0.043) |
| Breastfeeding |  |  |  |  |  |  |
| Never breastfed # | - | - | - |  |  |  |
| <2 months | -0.036(0.036) | -0.046(0.035) | -0.039(0.036) |  |  |  |
| 2.0-5.9 months | -0.102***(0.036) | -0.112***(0.040) | -0.095**(0.042) |  |  |  |
| ≥ 6 months | -0.175***(0.033) | -0.194***(0.034) | -0.131***(0.036) |  |  |  |
| **Child characteristics** |  |  |  |  |  |  |
| Limiting longstanding illness | 0.166**(0.080) | 0.225***(0.079) |  | 0.259***(0.064) |  |  |
| Child weight |  |  |  |  |  |  |
| Normal # |  |  |  |  |  | _ |
| Overweight |  |  |  |  |  | 0.063**(0.035) |
| Obese |  |  |  |  |  | 0.170***(0.050) |
| **Family socio-economic characteristics** |  |  |  |  |  |  |
| Lone parent |  | 0.220***(0.053) |  |  |  |  |
| Change in family structure |  |  |  |  |  |  |
| No change # |  | - |  |  |  |  |
| New partner |  | 0.182**(0.077) |  |  |  |  |
| Became single |  | 0.018(0.070) |  |  |  |  |
| Maternal education |  |  |  |  |  |  |
| NVQ Level 1&2 # | - | - | - | - | - | _ |
| NVQ Level 3 | -0.001(0.035) | -0.058(0.037) | -0.045(0.042) | -0.077*(0.042) | -0.075*(0.042) | -0.027(0.044) |
| NVQ Level 4&5 | -0.091***(0.028) | -0.116***(0.030) | -0.110***(0.032) | -0.150***(0.034) | -0.129***(0.034) | -0.069**(0.035) |
| None of these | 0.025(0.050) | 0.107**(0.053) | 0.140**(0.056) | 0.065(0.064) | -0.010(0.070) | 0.095(0.069) |
| **Mediating factors – Parental Stress** |  |  |  |  |  |  |
| Postpartum depression | 0.052**(0.023) |  | 0.084***(0.030) | 0.087***(0.030) | 0.046(0.029) |  |
| CPRS(standardised) | -0.512***(0.014) | -0.335***(0.014) | -0.308***(0.014) | -0.271***(0.014) | -0.234***(0.015) | -0.199***(0.015) |
| Maternal psychological distress in the previous survey | NA | 0.054(0.039) | 0.169***(0.040) | 0.244***(0.043) | 0.286***(0.035) | 0.251***(0.039) |
| Poor maternal general health |  | -0.052(0.040) | -0.120**(0.048) | -0.163***(0.048) | -0.123***(0.047) | 0.117*(0.064) |
| **Mediating factors - Parental Investment** | | | | | | |
| Childcare at 9 months |  |  |  |  |  |  |
| Parental care # |  |  | - | - | - | - |
| Formal care |  |  | 0.077*(0.040) | 0.123***(0.037) | 0.116***(0.037) | 0.097**(0.039) |
| Grandparent care |  |  | 0.049(0.038) | 0.082**(0.035) | 0.075**(0.036) | -0.023(0.034) |
| Other informal care |  |  | 0.017(0.070) | 0.007(0.087) | 0.040(0.078) | 0.014(0.066) |
| Housing tenure |  |  |  |  |  |  |
| Own/mortgaged # |  |  | - | - | - |  |
| Private rent |  |  | 0.080*(0.047) | 0.081*(0.048) | 0.141***(0.051) |  |
| Social rent |  |  | 0.093**(0.044) | 0.131***(0.049) | 0.165***(0.049) |  |
| Other |  |  | 0.087(0.096) | -0.122(0.104) | 0.060(0.098) |  |
| Reads to child |  |  |  | NA | NA | NA |
| Never/Occasionally # | - | - | - |  |  |  |
| Weekly | -0.130**(0.057) | -0.126(0.084) | -0.097*(0.052) |  |  |  |
| Daily | -0.199***(0.059) | -0.211**(0.081) | -0.147**(0.057) |  |  |  |
| Takes child to library |  | NA | NA | NA | NA | NA |
| Never | - |  |  |  |  |  |
| Occasionally | -0.127***(0.030) |  |  |  |  |  |
| Several times a week | -0.124***(0.029) |  |  |  |  |  |
| Tells story to child | NA |  |  | NA | NA | NA |
| Never/Occasionally |  | - |  |  |  |  |
| Weekly |  | -0.049*(0.027) |  |  |  |  |
| Daily |  | -0.079**(0.039) |  |  |  |  |
| Teaches painting | NA |  |  | NA | NA | NA |
| Never/Occasionally # |  | - |  |  |  |  |
| Weekly |  | -0.057**(0.029) |  |  |  |  |
| Daily |  | -0.127**(0.051) |  |  |  |  |
| Plays indoor games | NA |  |  |  | NA | NA |
| Never/Occasionally # |  |  | - |  |  |  |
| Weekly |  |  | -0.043(0.034) |  |  |  |
| Daily |  |  | -0.105**(0.050) |  |  |  |
| Talk things important to child | NA | NA | NA |  |  |  |
| Never/Occasionally |  |  |  | - | - | - |
| Weekly |  |  |  | -0.314***(0.100) | -0.175(0.140) | -0.336***(0.107) |
| Daily |  |  |  | -0.387***(0.100) | -0.255*(0.139) | -0.490***(0.108) |
| Home atmosphere | -0.049***(0.007) | -0.091***(0.006) | NA | NA | NA | NA |

Notes: marginal effects (standard error) reported; # reference group; * p<0.1 ** p<0.05 *** p<0.01; variables that were not available in a specific survey are shown as ‘NA’; variables that did not meet model selection criteria were dropped and are not reported.

|  |  |
| --- | --- |
|  |  |
|  |  |

## Figure A1. Permanent income vs. lagged transitory income on the association for ‘abnormal’ child mental health from 3 to 17 years. N=8096 up to 14; N=5667 at age 17

## Table A6. Complete case vs. main analysis at 3 years old

| Dependent variable: ‘abnormal’ overall mental health problems | | | | |  |
| --- | --- | --- | --- | --- | --- |
|  | Complete case | | Main analysis | | |
| Permanent income | **-0.014*** | | **-0.024***** | | |
|  | (0.086) | | (0.009) | | |
| Child health endowments |  | |  | | |
| Child age | -0.012 | | -0.029 | | |
|  | (0.552) | | (0.021) | | |
| Male child | 0.015** | | 0.016** | | |
|  | (0.047) | | (0.007) | | |
| Minority ethnic group | 0.049*** | | 0.046*** | | |
|  | (<0.001) | | (0.012) | | |
| Gestational age |  | |  | | |
| Term # | - | | - | | |
| Pre-term | -0.005 | | 0.011 | | |
|  | (0.704) | | (0.014) | | |
| Firstborn | 0.021*** | | 0.008 | | |
|  | (0.007) | | (0.009) | | |
| Pregnancy-related factors |  | |  | | |
| Maternal age at childbirth |  | |  | | |
| Less than 20 years | 0.032 | | 0.022 | | |
|  | (0.118) | | (0.020) | | |
| 20 to 24 years | 0.027** | | 0.036*** | | |
|  | (0.040) | | (0.014) | | |
| 25 to 29 years # | - | | - | | |
| 30 to 34 years | -0.006 | | -0.008 | | |
|  | (0.488) | | (0.008) | | |
| 35 or over | 0.005 | | -0.003 | | |
|  | (0.610) | | (0.009) | | |
| Breastfeeding |  | |  | | |
| Never breastfed # | - | | - | | |
| < 2 months | -0.017 | | -0.030*** | | |
|  | (0.136) | | (0.011) | | |
| 2.0-5.9 months | -0.034*** | | -0.055*** | | |
|  | (0.004) | | (0.013) | | |
| >=6 months | -0.043*** | | -0.056*** | | |
|  | (<0.001) | | (0.012) | | |
| Child characteristics |  | |  | | |
| Limiting physical longstanding illness | 0.044** | | 0.060*** | | |
|  | (0.020) | | (0.018) | | |
| Parental stress variables |  | |  | | |
| Postpartum depression | 0.008 | | 0.014* | | |
|  | (0.326) | | (0.008) | | |
| Child-parent relationship scale (standardised) | -0.066*** | | -0.076*** | | |
|  | (<0.001) | | (0.005) | | |
| Maternal poor general health | -0.018* | | 0.019** | | |
|  | (0.054) | | (0.009) | | |
| Parental investment variables |  | |  | | |
| Childcare at 9 months |  | |  | | |
| Parent care # | - | | - | | |
| Formal care | -0.027*** | | -0.017 | | |
|  | (0.007) | | (0.012) | | |
| Grandparent care | -0.012 | | -0.009 | | |
|  | (0.267) | | (0.012) | | |
| Other informal care | -0.044*** | | -0.048*** | | |
|  | (<0.001) | | (0.014) | | |
| Childcare at 3 years old |  | |  | | |
| Parent care # | - | | - | | |
| Formal care | -0.020** | | -0.020** | | |
|  | (0.038) | | (0.010) | | |
| Grandparent care | 0.012 | | 0.012 | | |
|  | (0.323) | | (0.013) | | |
| Other informal care | 0.008 | | 0.069 | | |
|  | (0.755) | | (0.052) | | |
| Reads to child |  | |  | | |
| Never/Occasionally # | - | | - | | |
| Weekly | -0.050** | | -0.047** | | |
|  | (0.016) | | (0.018) | | |
| Daily | -0.068*** | | -0.057*** | | |
|  | (0.002) | | (0.019) | | |
| Home atmosphere | -0.007*** | | -0.007*** | | |
|  | (<0.001) | | (0.002) | | |
| Number of observations | 6277 | 8096 | |  |  |
| Notes: marginal effects (standard error) reported; # reference group; * p<0.1 ** p<0.05 *** p<0.01; fully-adjusted model used. | | | | |  |

## Table A7. Complete case vs. main analysis at 5 years old

| Dependent variable: ‘abnormal’ overall mental health problems | | | |
| --- | --- | --- | --- |
|  | Complete case | Main analysis |  |
| Permanent income | **-0.008*** | **-0.014***** |  |
|  | (0.004) | (0.004) |  |
| Child health endowments |  |  |  |
| Child age | -0.005 | -0.001 |  |
|  | (0.006) | (0.007) |  |
| Male child | 0.014*** | 0.013*** |  |
|  | (0.003) | (0.004) |  |
| Minority ethnic group | 0.001 | 0.004 |  |
|  | (0.006) | (0.005) |  |
| Gestational age |  |  |  |
| Term # | - | - |  |
| Pre-term | -0.001 | <0.001 |  |
|  | (0.005) | (0.006) |  |
| Firstborn | 0.006 | 0.008** |  |
|  | (0.004) | (0.004) |  |
| Pregnancy-related factors |  |  |  |
| Maternal age at childbirth |  |  |  |
| Less than 20 years | 0.006 | 0.005 |  |
|  | (0.006) | (0.007) |  |
| 20 to 24 years | 0.016*** | 0.012** |  |
|  | (0.005) | (0.005) |  |
| 25 to 29 years # | - | - |  |
| 30 to 34 years | 0.002 | 0.003 |  |
|  | (0.003) | (0.004) |  |
| 35 or over | 0.008 | 0.006 |  |
|  | (0.005) | (0.006) |  |
| Breastfeeding |  |  |  |
| Never breastfed # | - | - |  |
| < 2 months | 0.002 | -0.001 |  |
|  | (0.004) | (0.004) |  |
| 2.0-5.9 months | <0.001 | <0.001 |  |
|  | (0.005) | (0.006) |  |
| >=6 months | -0.008* | -0.010** |  |
|  | (0.004) | (0.004) |  |
| Child characteristics |  |  |  |
| Limiting physical longstanding illness | 0.011 | 0.013* |  |
|  | (0.008) | (0.007) |  |
| Family socio-economic characteristics |  |  |  |
| Maternal education |  |  |  |
| NVQ Level 1&2 # | - | - |  |
| NVQ Level 3 | 0.006 | 0.005 |  |
|  | (0.005) | (0.005) |  |
| NVQ Level 4&5 | -0.001 | <0.001 |  |
|  | (0.004) | (0.004) |  |
| None of these | 0.005 | 0.011* |  |
|  | (0.006) | (0.006) |  |
| Parental stress variables |  |  |  |
| Postpartum depression | 0.007** | 0.010*** |  |
|  | (0.003) | (0.003) |  |
| Child-parent relationship scale (standardised) | -0.012*** | -0.014*** |  |
|  | (0.002) | (0.002) |  |
| Maternal psychological distress in the previous survey | 0.009*** | 0.010*** |  |
|  | (0.003) | (0.004) |  |
| Parental investment variables |  |  |  |
| Childcare at 9 months |  |  |  |
| Parent care # | - | - |  |
| Formal care | -0.009** | -0.009* |  |
|  | (0.004) | (0.005) |  |
| Grandparent care | 0.003 | 0.002 |  |
|  | (0.005) | (0.005) |  |
| Other informal care | -0.015*** | -0.016*** |  |
|  | (0.004) | (0.006) |  |
| Reads to child |  |  |  |
| Never/Occasionally # | - | - |  |
| Weekly | -0.026** | -0.028** |  |
|  | (0.012) | (0.013) |  |
| Daily | -0.035*** | -0.036*** |  |
|  | (0.012) | (0.013) |  |
| Teaches painting |  |  |  |
| Never/Occasionally # | - | - |  |
| Weekly | -0.003 | -0.006 |  |
|  | (0.003) | (0.004) |  |
| Daily | -0.007 | -0.013** |  |
|  | (0.005) | (0.005) |  |
| Home atmosphere | -0.003*** | -0.004*** |  |
|  | (0.001) | (0.001) |  |
| Number of observations | 6683 | 8096 |  |
| Notes: marginal effects (standard error) reported; # reference group; * p<0.1 ** p<0.05 *** p<0.01; fully-adjusted model used. | | |  |

## Table A8. Complete case vs. main analysis at 7 years old

| Dependent variable: ‘abnormal’ overall mental health problems | | | | |
| --- | --- | --- | --- | --- |
|  | Complete case | Main analysis | |  |
| Permanent income | **-0.011** | **-0.009** | |  |
|  | (0.007) | (0.006) | |  |
| Child health endowments |  |  | |  |
| Child age | -0.006 | -0.003 | |  |
|  | (0.010) | (0.009) | |  |
| Male child | 0.021*** | 0.026*** | |  |
|  | (0.005) | (0.005) | |  |
| Minority ethnic group | 0.006 | 0.001 | |  |
|  | (0.009) | (0.007) | |  |
| Gestational age |  |  | |  |
| Term # | - | - | |  |
| Pre-term | 0.002 | -0.002 | |  |
|  | (0.010) | (0.010) | |  |
| Firstborn | 0.008* | 0.007 | |  |
|  | (0.005) | (0.005) | |  |
| Pregnancy-related factors |  |  | |  |
| Breastfeeding |  |  | |  |
| Never breastfed # | - | - | |  |
| < 2 months | 0.006 | 0.004 | |  |
|  | (0.007) | (0.007) | |  |
| 2.0-5.9 months | -0.012* | -0.012* | |  |
|  | (0.007) | (0.007) | |  |
| >=6 months | -0.019*** | -0.020*** | |  |
|  | (0.007) | (0.006) | |  |
| Child characteristics |  |  | |  |
| Limiting physical longstanding illness | 0.020* | 0.025** | |  |
|  | (0.010) | (0.010) | |  |
| Parental stress variables |  |  | |  |
| Postpartum depression | 0.013** | 0.014*** | |  |
|  | (0.006) | (0.005) | |  |
| Child-parent relationship scale (standardised) | -0.022*** | -0.025*** | |  |
|  | (0.002) | (0.002) | |  |
| Maternal psychological distress in the previous survey | 0.014** | 0.022*** | |  |
|  | (0.005) | (0.006) | |  |
| Maternal poor general health | -0.026*** | 0.025*** | |  |
|  | (0.007) | (0.006) | |  |
| Parental investment variables |  |  | |  |
| Housing tenure |  |  | |  |
| Own/mortgaged # | - | - | |  |
| Private rent | 0.023** | 0.022** | |  |
|  | (0.011) | (0.011) | |  |
| Social rent | 0.025*** | 0.025*** | |  |
|  | (0.009) | (0.008) | |  |
| Other | -0.014 | -0.008 | |  |
|  | (0.015) | (0.015) | |  |
| Reads to child |  |  | |  |
| Never/Occasionally # | - | - | |  |
| Weekly | -0.020* | -0.027** | |  |
|  | (0.011) | (0.011) | |  |
| Daily | -0.026** | -0.026** | |  |
|  | (0.011) | (0.012) | |  |
| Goes to parks |  |  | |  |
| Never/Occasionally # | - | - | |  |
| Weekly | -0.009* | -0.009* | |  |
|  | (0.005) | (0.005) | |  |
| Daily | 0.006 | 0.009 | |  |
|  | (0.017) | (0.016) | |  |
| Teaches painting |  |  | |  |
| Never/Occasionally # | - | - | |  |
| Weekly | 0.009 | 0.011* | |  |
|  | (0.006) | (0.006) | |  |
| Daily | 0.025 | 0.018 | |  |
|  | (0.020) | (0.016) | |  |
| Number of observations | 6545 | 8096 | |  |
| Notes: marginal effects (standard error) reported; # reference group; * p<0.1 ** p<0.05 *** p<0.01; fully-adjusted model used. | | |  |  |

## Table A9. Complete case vs. main analysis at 11 years old

| Dependent variable: ‘abnormal’ overall mental health problems | | |  |
| --- | --- | --- | --- |
|  | Complete case | Main analysis | |
| Permanent income | **-0.043***** | **-0.048***** | |
|  | (0.010) | (0.010) | |
| Child health endowments |  |  | |
| Child age | -0.017 | -0.022** | |
|  | (0.011) | (0.010) | |
| Male child | 0.027*** | 0.036*** | |
|  | (0.007) | (0.007) | |
| Minority ethnic group | -0.031** | -0.030** | |
|  | (0.015) | (0.014) | |
| Gestational age |  |  | |
| Term # | - | - | |
| Pre-term | -0.009 | 0.010 | |
|  | (0.010) | (0.011) | |
| Firstborn | 0.015** | 0.013* | |
|  | (0.007) | (0.007) | |
| Pregnancy-related factors |  |  | |
| Maternal alcohol consumption during pregnancy |  |  | |
| Never # | - | - | |
| Light | -0.014* | -0.019** | |
|  | (0.008) | (0.008) | |
| Moderate/Heavy | 0.012 | 0.016 | |
|  | (0.014) | (0.013) | |
| Breastfeeding |  |  | |
| Never breastfed # | - | - | |
| < 2 months | 0.020** | 0.021** | |
|  | (0.009) | (0.008) | |
| 2.0-5.9 months | -0.008 | -0.003 | |
|  | (0.009) | (0.011) | |
| >=6 months | -0.006 | -0.001 | |
|  | (0.009) | (0.009) | |
| Child characteristics |  |  | |
| Limiting physical longstanding illness | 0.023* | 0.020* | |
|  | (0.012) | (0.012) | |
| Child weight |  |  | |
| Normal # | - | - | |
| Overweight | 0.023** | 0.017* | |
|  | (0.011) | (0.010) | |
| Obese | 0.022 | 0.016 | |
|  | (0.015) | (0.014) | |
| Parental stress variables |  |  | |
| Postpartum depression | 0.007 | 0.018** | |
|  | (0.007) | (0.007) | |
| Child-parent relationship scale (standardised) | -0.024*** | -0.027*** | |
|  | (0.003) | (0.003) | |
| Maternal psychological distress in the previous survey | 0.042*** | 0.041*** | |
|  | (0.008) | (0.008) | |
| Maternal poor general health | -0.034*** | 0.023** | |
|  | (0.010) | (0.009) | |
| Parental investment variables |  |  | |
| Housing tenure |  |  | |
| Own/mortgaged # | - | - | |
| Private rent | 0.013 | 0.016 | |
|  | (0.012) | (0.012) | |
| Social rent | 0.009 | 0.007 | |
|  | (0.012) | (0.011) | |
| Other | -0.051*** | -0.042*** | |
|  | (0.007) | (0.013) | |
| Plays indoor games |  |  | |
| Never/Occasionally # | - | - | |
| Weekly | 0.008 | 0.012* | |
|  | (0.008) | (0.007) | |
| Daily | -0.004 | 0.002 | |
|  | (0.018) | (0.017) | |
| Number of observations | 6410 | 8096 | |
| Notes: marginal effects (standard error) reported; # reference group; * p<0.1 ** p<0.05 *** p<0.01; fully-adjusted model used. | | | |

## Table A10. Complete case vs. main analysis at 14 years old

| Dependent variable: ‘abnormal’ overall mental health problems | | |  |
| --- | --- | --- | --- |
|  | Complete case | Main analysis | |
| Permanent income | **-0.032***** | **-0.042***** | |
|  | (0.012) | (0.011) | |
| Child health endowments |  |  | |
| Child age | -0.010 | -0.010 | |
|  | (0.013) | (0.012) | |
| Male child | 0.030*** | 0.025*** | |
|  | (0.008) | (0.007) | |
| Minority ethnic group | -0.012 | -0.023** | |
|  | (0.016) | (0.011) | |
| Gestational age |  |  | |
| Term # |  |  | |
| Pre-term | 0.001 | 0.015 | |
|  | (0.015) | (0.012) | |
| Firstborn | 0.003 | -0.006 | |
|  | (0.008) | (0.008) | |
| Pregnancy-related factors |  |  | |
| Maternal alcohol consumption during pregnancy |  |  | |
| Never # | - | - | |
| Light | -0.003 | -0.003 | |
|  | (0.009) | (0.009) | |
| Moderate/Heavy | 0.037* | 0.034** | |
|  | (0.019) | (0.016) | |
| Breastfeeding |  |  | |
| Never breastfed # | - | - | |
| < 2 months | 0.013 | 0.024*** | |
|  | (0.010) | (0.009) | |
| 2.0-5.9 months | 0.003 | 0.002 | |
|  | (0.011) | (0.011) | |
| >=6 months | -0.002 | -0.003 | |
|  | (0.011) | (0.010) | |
| Child characteristics |  |  | |
| Child weight |  |  | |
| Normal # | - | - | |
| Overweight | 0.029** | 0.025** | |
|  | (0.011) | (0.010) | |
| Obese | 0.036** | 0.036** | |
|  | (0.018) | (0.016) | |
| Parental stress variables |  |  | |
| Child-parent relationship scale (standardised) | -0.029*** | -0.032*** | |
|  | (0.004) | (0.004) | |
| Maternal psychological distress in the previous survey | 0.051*** | 0.052*** | |
|  | (0.008) | (0.008) | |
| Maternal poor general health | -0.024** | 0.028*** | |
|  | (0.010) | (0.009) | |
| Parental investment variables |  |  | |
| Housing tenure |  |  | |
| Own/mortgaged # | - | - | |
| Private rent | 0.020 | 0.030** | |
|  | (0.014) | (0.013) | |
| Social rent | 0.028* | 0.018 | |
|  | (0.016) | (0.013) | |
| Other | -0.007 | -0.015 | |
|  | (0.021) | (0.018) | |
| Number of observations | 6000 | 8096 | |
| Notes: marginal effects (standard error) reported; # reference group; * p<0.1 ** p<0.05 *** p<0.01; fully-adjusted model used. | | | |

## Table A11. Complete case vs. main analysis at 17 years old

| Dependent variable: ‘abnormal’ overall mental health problems | | |
| --- | --- | --- |
|  | Complete case | Main analysis |
| Permanent income | **-0.022**** | **-0.042***** |
|  | (0.011) | (0.008) |
| **Child health endowments** |  |  |
| Child age | -0.004 | -0.003 |
|  | (0.011) | (0.010) |
| Male child | -0.002 | 0.011 |
|  | (0.008) | (0.007) |
| Minority ethnic group | -0.005 | -0.014 |
|  | (0.016) | (0.010) |
| Gestational age | -0.002 | 0.006 |
|  | (0.016) | (0.012) |
| Firstborn | -0.004 | -0.003 |
|  | (0.008) | (0.007) |
| **Child characteristics** |  |  |
| Limiting physical longstanding illness | 0.036*** | 0.020 |
|  | (0.014) | (0.015) |
| Child weight |  |  |
| Normal # | - | - |
| Overweight | 0.004 | 0.009 |
|  | (0.010) | (0.008) |
| Obese | 0.047** | 0.043*** |
|  | (0.019) | (0.014) |
| **Family socio-economic characteristics** |  |  |
| Lone-parent | 0.011 | 0.020*** |
|  | (0.010) | (0.008) |
| Change in family structure |  |  |
| No change # | - | - |
| New partner | 0.000 | -0.043*** |
|  | (.) | (0.011) |
| Became single | -0.016 | -0.003 |
|  | (0.013) | (0.012) |
| **PS variables** |  |  |
| Postpartum depression | 0.016* | 0.012* |
|  | (0.008) | (0.007) |
| Child-parent relationship scale (standardised) | -0.010*** | -0.017*** |
|  | (0.004) | (0.003) |
| Maternal psychological distress in the previous sweep | 0.035*** | 0.038*** |
|  | (0.009) | (0.007) |
| Maternal self-reported general health | 0.028*** | 0.031*** |
|  | (0.010) | (0.010) |
| Number of observations | 2738 | 5667 |
| Notes: marginal effects (standard error) reported; # reference group; * p<0.1 ** p<0.05 *** p<0.01; fully-adjusted model used. | | |

|  |  |
| --- | --- |
|  |  |
|  |  |

## **Figure A2. Association between permanent income and ‘abnormal’ child mental health using main and restricted sample from 3 to 17 years.** Main sample: N=8,096 at age 3, 5, 7, 11, and 14, N=5,667 at age 17; Restricted sample: N=5,667 from age 3 to 17.

## Table A12. Descriptive statistics of available cases and missing data

| Variables (N (%)) | 3 years | 5 years | 7 years | 11 years | 14 years | 17 years |
| --- | --- | --- | --- | --- | --- | --- |
|  | N=8096 | | | | | N=5667 |
| **Child mental health** |  |  |  |  |  |  |
| ‘Abnormal’ mental health symptoms (TDS) | 895(14.1) | 373(5.8) | 492(7.1) | 610(9.5) | 681(10.2) | 454(7.9) |
| *Missing* | *391(4.8)* | *200(2.5)* | *174(2.1)* | *197(2.4)* | *232(2.9)* | *49(0.9)* |
| Total Difficulties Score (Mean(SD)) | 9.6 (5.2) | 7.3 (5.0) | 7.5 (5.4) | 7.9 (5.9) | 8.2 (6.1) | 7.0 (5.8) |
| *Missing* | 391 (4.8) | *200 (2.5)* | 174 (2.1) | *197 (2.4)* | 232 (2.9) | *49 (0.9)* |
| Internalising problems (Mean(SD)) | 2.9 (2.4) | 2.5 (2.5) | 2.8 (2.8) | 3.3 (3.2) | 3.8 (3.5) | 3.7 (3.5) |
| *Missing* | *313 (3.9)* | *174(2.1)* | *157(1.9)* | *187(2.3)* | *225(2.8)* | *42(0.7)* |
| Externalising problems (Mean(SD)) | 6.8 (3.8) | 4.8 (3.5) | 4.8 (3.6) | 4.6 (3.6) | 4.4 (3.6) | 3.4 (3.2) |
| *Missing* | *316(3.9)* | *174(2.1)* | *148(1.8)* | *193(2.4)* | *229(2.8)* | *44(0.8)* |
| **Family income** |  |  |  |  |  |  |
| Permanent income (£) (Mean) | 25151 | 24766 | 24402 | 24495 | 24361 | 28726 |
| Permanent income (£) (SD) | 15305 | 14327 | 13316 | 12212 | 11470 | 9979 |
| *Missing* | *142(2.8)* | *174(2.1)* | *253(3.1)* | *253(3.1)* | *262(3.2)* | *2233(39.4)* |
| Lagged transitory income (£) (Mean) | 25768 | 24950 | 24361 | 24484 | 25134 | 27137 |
| Lagged transitory income (£) (SD) | 16714 | 16597 | 14997 | 14240 | 10537 | 9837 |
| *Missing* | *86(1.1)* | *69(0.9)* | *56(0.7)* | *125(1.5)* | *0* | *5(0.1)* |
| **Child health endowments** |  |  |  |  |  |  |
| Child age, years (Mean(SD)) | 3.1(0.2) | 5.2(0.2) | 7.2(0.2) | 11.2(0.3) | 14.3(0.3) | 17.2(0.3) |
| *Missing* | *2(<0.1)* | *0* | *3(<0.1)* | *0* | *14(0.2)* | *0* |
| Child sex |  |  |  |  |  |  |
| Female | 4104(49.5) | " | " | " | " | 2930(51.1) |
| Male | 3992(50.5) | " | " | " | " | 2737(48.9) |
| *Missing* | *0* | " | " | " | " | *0* |
| Child ethnicity |  |  |  |  |  |  |
| White | 7074(87.9) | " | " | " | " | 4966(92.0) |
| Minority ethnic group | 1022(12.1) | " | " | " | " | 701(8.0) |
| *Missing* | *0* | " | " | " | " | *0* |
| Gestational age at birth |  |  |  |  |  |  |
| Preterm (<37 weeks gestation) | 533(7.4) | " | " | " | " | 376(7.1) |
| Term (> 37 weeks at gestation) | 7213(92.6) | " | " | " | " | 5047(92.9) |
| *Missing* | *350(4.3)* | " | " | " | " | 244(4.3) |
| Firstborn | 4064(51.1) | " | " | " | " | 2893(51.8) |
| *Missing* | *0* | " | " | " | " | *0* |
| **Pregnancy-related factors** |  |  |  |  |  |  |
| Maternal age at childbirth* |  |  |  |  |  |  |
| Less than 20 years | 414(7.9) | " | " | " | " | 258(3.7) |
| 20 to 24 years | 1164(16.7) | " | " | " | " | 781(11.2) |
| 25 to 29 years | 2264(28.8) | " | " | " | " | 1590(27.8) |
| 30 to 34 years | 2726(29.9) | " | " | " | " | 1951(36.2) |
| 35 or over | 1525(16.7) | " | " | " | " | 1086(21.1) |
| *Missing* | *3(<0.1)* | " | " | " | " | *1(<0.1)* |
| Maternal smoking during pregnancy* |  |  |  |  |  |  |
| Never smoked | 5655(65.4) | " | " | " | " | 3987(71.9) |
| Stopped smoking during pregnancy | 949(12.8) | " | " | " | " | 663(12.2) |
| Smoked throughout pregnancy | 1480(21.8) | " | " | " | " | 1009(15.9) |
| *Missing* | *12(0.1)* | " | " | " | " | *8(0.1)* |
| Maternal alcohol consumption during pregnancy |  |  |  |  |  |  |
| Never | 5436(67.7) | " | " | " | " | 3765(62.7) |
| Light | 2055(25.1) | " | " | " | " | 1482(29.9) |
| Moderate/Heavy | 567(7.2) | " | " | " | " | 396(7.4) |
| *Missing* | *38(0.5)* | " | " | " | " | *24(0.4)* |
| Breastfeeding* |  |  |  |  |  |  |
| Never breastfed | 2216(33.6) | " | " | " | " | 1448(22.7) |
| <2 months | 2144(24.2) | " | " | " | " | 1486(25.1) |
| 2.0-5.9 months | 1640(19) | " | " | " | " | 1160(21.7) |
| ≥ 6 months | 2093(23.2) | " | " | " | " | 1572(30.5) |
| *Missing* | *3(<0.1)* | " | " | " | " | *1(<0.1)* |
| **Child characteristics** |  |  |  |  |  |  |
| Child with limiting longstanding illness | 210(3) | 396(5) | 413(5.4) | 368(4.8) | 408(4.9) | 242(4.2) |
| *Missing* | *19(0.2)* | *20(0.2)* | *21(0.3)* | *44(0.5)* | *56(0.7)* | *211(3.5)* |
| Child weight |  |  |  |  |  |  |
| Normal | 5773(77.3) | 6355(79.2) | 6448(80.4) | 5791(72.9) | 5684(73.7) | 3937(72.3) |
| Overweight | 1352(17.3) | 1265(15.8) | 1129(14.3) | 1638(21) | 1470(19.2) | 1047(18.7) |
| Obese | 411(5.4) | 405(5) | 437(5.3) | 486(6.1) | 515(7.1) | 524(9.1) |
| *Missing* | *560(6.9)* | *71(0.9)* | *82(1)* | *181(2.2)* | *427(5.3)* | *458(7.6)* |
| **Family socio-economic characteristics** |  |  |  |  |  |  |
| Lone parent | 1052(17.3) | 1262(20.2) | 1404(21.4) | 1640(24.8) | 1750(25.8) | 1571(25.8) |
| *Missing* | *11(0.1)* | *0* | *0* | *0* | *0* | *13(0.2)* |
| Change in family structure* |  |  |  |  |  |  |
| No change | 7314(88.4) | 7425(89.9) | 7470(90.1) | 7195(86.9) | 5345(94.3) | 5646(94.3) |
| New partner | 366(5.3) | 256(4) | 253(4.5) | 376(5.5) | 30(0.5) | 30(0.5) |
| Became single | 405(6.3) | 404(6.1) | 373(5.4) | 525(7.6) | 291(5.2) | 309(5.3) |
| *Missing* | *11(0.1)* | *11(0.1)* | *0* | *0* | *1(<0.1)* | *14(0.2)* |
| Maternal education |  |  |  |  |  |  |
| NVQ Level 1&2 | 2753(38.6) | 2646(37.5) | 2534(36.1) | 2367(34.3) | 2227(32.3) | 1582(26.9) |
| NVQ Level 3 | 1234(14.3) | 1244(14.6) | 1262(15.1) | 1227(15) | 1204(14.8) | 885(14.2) |
| NVQ Level 4&5 | 3196(31.9) | 3346(33.5) | 3493(35.1) | 3750(38.1) | 3965(41.2) | 3036(52.8) |
| None of these | 913(15.2) | 860(14.4) | 807(13.7) | 752(12.6) | 700(11.7) | 496(6.1) |
| *Missing* | *0* | *0* | *0* | *0* | *0* | *0* |
| Notes: Unweighted counts (N) and survey-weighted proportion of non-missing values (%) reported; SD standard deviation. | | | | | | |

## Table A13. Baseline characteristics of complete cases vs. incomplete cases

|  | 3 years |  |  | 5 years |  |  | 7 | years |  | 11 years |  |  | 14 years |  |  | 17 years |  |  |
| --- | --- | --- | --- | --- | --- | --- | --- | --- | --- | --- | --- | --- | --- | --- | --- | --- | --- | --- |
| Characteristic | Comp (n=6277) | Incomp (n=1819) | *p* | Comp (n=6683) | Incomp (n=1413) | *p* | Comp (n=6545) | Incomp (n=1551) | *p* | Comp (n=6410) | Incomp (n=1686) | *p* | Comp (n=6000) | Incomp (n=2096) | *p* | Comp (n=2722) | Incomp (n=2945) | *p* |
| ‘Abnormal’ mental health (%) | 10.9 | 14.9 | <0.001 | 4.2 | 7.7 | <0.001 | 5.9 | 7.8 | 0.006 | 7.3 | 9.5 | 0.005 | 8 | 10.7 | <0.001 | 5.9 | 10.1 | <0.001 |
| Permanent income (mean) | 27912 | 22888 | <0.001 | 27360 | 21170 | <0.001 | 26889 | 21016 | <0.001 | 26808 | 21636 | <0.001 | 26690 | 22172 | <0.001 | 27713 | 25339 | <0.001 |
| Ln(permanent income)(mean) | 10.08 | 9.83 | <0.001 | 10.07 | 9.77 | <0.001 | 10.07 | 9.78 | <0.001 | 10.09 | 9.83 | <0.001 | 10.1 | 9.87 | <0.001 | 10.16 | 10.05 | <0.001 |
| Child age (mean) | 3.12 | 3.13 | 0.013 | 5.2 | 5.21 | 0.258 | 7.22 | 7.23 | 0.384 | 11.15 | 11.16 | 0.751 | 14.25 | 14.25 | 0.692 | 17.15 | 17.17 | 0.01 |
| Male child (%) | 48.7 | 51.3 | 0.048 | 49.4 | 49 | 0.827 | 49.4 | 49.1 | 0.831 | 49.3 | 49.3 | 0.971 | 50.3 | 46.4 | 0.002 | 47.5 | 49 | 0.249 |
| Minority ethnic group (%) | 8.9 | 25.5 | <0.001 | 8.7 | 31.4 | <0.001 | 8.4 | 30.3 | <0.001 | 8.5 | 28.1 | <0.001 | 8.5 | 24.5 | <0.001 | 6.8 | 17.5 | <0.001 |
| Firstborn (%) | 49.9 | 51.2 | 0.314 | 49.7 | 52.5 | 0.055 | 49.8 | 51.8 | 0.151 | 49.8 | 51.8 | 0.144 | 50.3 | 50 | 0.794 | 50.6 | 51.5 | 0.47 |
| Pre-term (%) | 6.6 | 8.1 | 0.04 | 6.9 | 7.3 | 0.526 | 6.7 | 7.8 | 0.159 | 6.7 | 7.6 | 0.271 | 6.8 | 7.3 | 0.399 | 6.2 | 7.7 | 0.035 |
| Maternal age (%) |  |  | 0.01 |  |  | <0.001 |  |  | <0.001 |  |  | 0.003 |  |  | 0.078 |  |  | <0.001 |
| Less than 20 | 5 | 5.4 |  | 5.1 | 5.2 |  | 5.3 | 4.4 |  | 5.2 | 4.6 |  | 5.1 | 5.1 |  | 3.1 | 5.9 |  |
| 20 to 24 years | 14 | 15.9 |  | 13.8 | 17.3 |  | 13.7 | 17.2 |  | 13.7 | 16.8 |  | 13.8 | 16 |  | 11.2 | 16.2 |  |
| 25-29 years | 27.6 | 29.3 |  | 27.8 | 29 |  | 28.1 | 27.6 |  | 28 | 28 |  | 27.9 | 28.3 |  | 28.4 | 27.8 |  |
| 30 to 34 years | 34.7 | 30.3 |  | 34.7 | 28.9 |  | 34.4 | 30.6 |  | 34.4 | 30.8 |  | 34.4 | 31.7 |  | 37.2 | 31.9 |  |
| 35 or over | 18.8 | 19.1 |  | 18.7 | 19.6 |  | 18.5 | 20.2 |  | 18.6 | 19.7 |  | 18.8 | 19 |  | 20.1 | 18.3 |  |
| Maternal smoking during pregnancy (%) |  |  | 0.01 |  |  | <0.001 |  |  | <0.001 |  |  | <0.001 |  |  | 0.007 |  |  | <0.001 |
| Never smoked | 69.1 | 72.8 |  | 68.9 | 75.2 |  | 68.6 | 75.8 |  | 68.9 | 73.8 |  | 69 | 72.6 |  | 73.2 | 67.9 |  |
| Stopped smoking during pregnancy | 12.2 | 10.3 |  | 12.2 | 9.8 |  | 12.1 | 10 |  | 12.1 | 10.5 |  | 12.2 | 10.3 |  | 12.1 | 11.4 |  |
| Smoked throughout pregnancy | 18.7 | 16.9 |  | 19 | 15.1 |  | 19.3 | 14.2 |  | 19 | 15.7 |  | 18.7 | 17.1 |  | 14.7 | 20.7 |  |
| Maternal alcohol consumption during pregnancy (%) |  |  | 0.002 |  |  | <0.001 |  |  | <0.001 |  |  | <0.001 |  |  | <0.001 |  |  | <0.001 |
| Never | 66.5 | 71 |  | 66.2 | 73.7 |  | 66.4 | 72 |  | 66.3 | 72 |  | 65.9 | 72.1 |  | 63.4 | 69.8 |  |
| Light | 26.3 | 22.6 |  | 26.5 | 20.7 |  | 26.3 | 21.9 |  | 26.3 | 22.3 |  | 26.7 | 22 |  | 30.2 | 22.6 |  |
| Moderate/Heavy | 7.2 | 6.4 |  | 7.3 | 5.6 |  | 7.3 | 6.1 |  | 7.4 | 5.7 |  | 7.4 | 5.9 |  | 6.4 | 7.6 |  |
| Breastfeeding (%) |  |  | 0.243 |  |  | 0.153 |  |  | 0.263 |  |  | 0.434 |  |  | 0.497 |  |  | 0.002 |
| Never breastfed | 27.8 | 26.1 |  | 27.5 | 26.7 |  | 27.7 | 26 |  | 27.6 | 26.4 |  | 27.2 | 28 |  | 23.3 | 27.6 |  |
| <2 months | 26.2 | 27.5 |  | 26.3 | 27.6 |  | 26.4 | 26.8 |  | 26.5 | 26.4 |  | 26.5 | 26.5 |  | 26.6 | 25.9 |  |
| 2.0-5.9 months | 20.5 | 19.4 |  | 20.6 | 18.4 |  | 20.4 | 19.6 |  | 20.4 | 19.8 |  | 20.6 | 19.2 |  | 20.8 | 20.2 |  |
| ≥ 6 months | 25.6 | 26.9 |  | 25.6 | 27.3 |  | 25.5 | 27.6 |  | 25.5 | 27.3 |  | 25.7 | 26.4 |  | 29.3 | 26.3 |  |
| Limiting longstanding illness (%) | 2.5 | 3 | 0.226 | 4.6 | 6.2 | 0.016 | 4.9 | 6.1 | 0.057 | 4.6 | 4.4 | 0.787 | 5 | 5.2 | 0.685 | 3.9 | 4.6 | 0.223 |
| Child weight (%) |  |  | 0.27 |  |  | 0.096 |  |  | 0.313 |  |  | 0.93 |  |  | 0.846 |  |  | 0.706 |
| Not overweight | 76.4 | 77.7 |  | 79.1 | 79.4 |  | 80.8 | 79 |  | 73.2 | 72.8 |  | 74 | 74.5 |  | 71.5 | 70.6 |  |
| Overweight | 18.2 | 16.4 |  | 16 | 14.5 |  | 13.9 | 15.1 |  | 20.7 | 20.9 |  | 19.2 | 19.1 |  | 18.8 | 19.1 |  |
| Obese | 5.4 | 5.9 |  | 4.8 | 6 |  | 5.4 | 5.9 |  | 6.1 | 6.3 |  | 6.8 | 6.4 |  | 9.7 | 10.3 |  |
| Lone parent (%) | 12.1 | 16 | <0.001 | 14.9 | 18.6 | 0.001 | 17.1 | 18.2 | 0.295 | 19.8 | 21.9 | 0.061 | 20.5 | 24.8 | <0.001 | 24.2 | 29.1 | <0.001 |
| Change in family structure (%) |  |  |  |  |  | 0.713 |  |  | 0.745 |  |  | 0.338 |  |  | 0.246 |  |  | 0.478 |
| No change | 90.9 | 89 | 0.046 | 91.7 | 92.3 |  | 92.2 | 92.5 |  | 88.7 | 89.4 |  | 91.5 | 90.4 |  | 94.7 | 94 |  |
| New partner | 4.4 | 4.9 |  | 3.2 | 3.1 |  | 3.1 | 3.2 |  | 4.8 | 4 |  | 3.9 | 4.1 |  | 0.4 | 0.6 |  |
| Became single | 4.7 | 6 |  | 5.1 | 4.6 |  | 4.7 | 4.3 |  | 6.5 | 6.6 |  | 4.7 | 5.5 |  | 4.9 | 5.4 |  |
| Maternal education (%) |  |  | <0.001 |  |  | <0.001 |  |  | <0.001 |  |  | <0.001 |  |  | <0.001 |  |  | <0.001 |
| NVQ Level 1&2 | 35 | 30.6 |  | 33.5 | 28.7 |  | 32.3 | 27.1 |  | 29.9 | 26.9 |  | 27.4 | 27.8 |  | 23.4 | 29.7 |  |
| NVQ Level 3 | 16.1 | 12.1 |  | 16.3 | 11.1 |  | 16.5 | 11.7 |  | 15.9 | 12.2 |  | 15.4 | 13.4 |  | 16.2 | 13.4 |  |
| NVQ Level 4&5 | 41.1 | 34 |  | 42.8 | 34.2 |  | 44.5 | 37.3 |  | 48.1 | 39.6 |  | 51.7 | 41.2 |  | 57.2 | 45.4 |  |
| None of these | 7.8 | 23.3 |  | 7.4 | 26 |  | 6.7 | 23.8 |  | 6.1 | 21.4 |  | 5.5 | 17.7 |  | 3.3 | 11.5 |  |
| **p* values from t-test for continuous variables and chi-squared test for categorical variables. | | | | | | | | | | | | | | | | | | |

## Reference

1. Boyle, E. M., Poulsen, G., Field, D. J., Kurinczuk, J. J., Wolke, D., Alfirevic, Z., & Quigley, M. A. (2012). Effects of gestational age at birth on health outcomes at 3 and 5 years of age: population based cohort study. BMJ, 344.
2. Driscoll, K., & Pianta, R. C. (2011). Mothers' and fathers' perceptions of conflict and closeness in parent-child relationships during early childhood. Journal of Early Childhood and Infant Psychology, (7), 1-24.
3. Ramasubramanian, L., Lane, S., & Rahman, A. (2013). The association between maternal serious psychological distress and child obesity at 3 years: a cross‐sectional analysis of the UK Millennium Cohort Data. Child: care, health and development, 39(1), 134-140.
